# High-Resolution Mapping of Transcription Initiation in the Asexual Stages of *Toxoplasma gondii*

**DOI:** 10.3389/fcimb.2020.617998

**Published:** 2021-01-20

**Authors:** Benedikt M. Markus, Benjamin S. Waldman, Hernan A. Lorenzi, Sebastian Lourido

**Affiliations:** ^1^ Whitehead Institute for Biomedical Research, Cambridge, MA, United States; ^2^ Faculty of Biology, University of Freiburg, Freiburg, Germany; ^3^ Department of Biology, Massachusetts Institute of Technology, Cambridge, MA, United States; ^4^ J. Craig Venter Institute, Rockville, MD, United States

**Keywords:** Apicomplexa, transcription start site mapping, 5′-end RNA sequencing, 5′ untranslated regions, uORF, nucleosome positioning, transcriptional regulation, core-promoter elements

## Abstract

*Toxoplasma gondii* is a common parasite of humans and animals, causing life-threatening disease in the immunocompromized, fetal abnormalities when contracted during gestation, and recurrent ocular lesions in some patients. Central to the prevalence and pathogenicity of this protozoan is its ability to adapt to a broad range of environments, and to differentiate between acute and chronic stages. These processes are underpinned by a major rewiring of gene expression, yet the mechanisms that regulate transcription in this parasite are only partially characterized. Deciphering these mechanisms requires a precise and comprehensive map of transcription start sites (TSSs); however, *Toxoplasma* TSSs have remained incompletely defined. To address this challenge, we used 5′-end RNA sequencing to genomically assess transcription initiation in both acute and chronic stages of *Toxoplasma*. Here, we report an in-depth analysis of transcription initiation at promoters, and provide empirically-defined TSSs for 7603 (91%) protein-coding genes, of which only 1840 concur with existing gene models. Comparing data from acute and chronic stages, we identified instances of stage-specific alternative TSSs that putatively generate mRNA isoforms with distinct 5′ termini. Analysis of the nucleotide content and nucleosome occupancy around TSSs allowed us to examine the determinants of TSS choice, and outline features of *Toxoplasma* promoter architecture. We also found pervasive divergent transcription at *Toxoplasma* promoters, clustered within the nucleosomes of highly-symmetrical phased arrays, underscoring chromatin contributions to transcription initiation. Corroborating previous observations, we asserted that *Toxoplasma* 5′ leaders are among the longest of any eukaryote studied thus far, displaying a median length of approximately 800 nucleotides. Further highlighting the utility of a precise TSS map, we pinpointed motifs associated with transcription initiation, including the binding sites of the master regulator of chronic-stage differentiation, BFD1, and a novel motif with a similar positional arrangement present at 44% of *Toxoplasma* promoters. This work provides a critical resource for functional genomics in *Toxoplasma*, and lays down a foundation to study the interactions between genomic sequences and the regulatory factors that control transcription in this parasite.

## Introduction

A precise map of transcription start sites (TSSs) is indispensable for identifying promoters and other regulatory factors that mediate gene expression in an organism. Quantitative maps of transcription initiation have revealed the complex and dynamic nature of transcription initiation landscapes in a range of model eukaryotes, including yeast, flies, worms, zebrafish, and mammals (e.g., Carninci et al., 2006; Hoskins et al., 2011; Chen et al., 2013; FANTOM Consortium and the RIKEN PMI and CLST (DGT) et al., 2014; Haberle et al., 2014; Lu and Lin, 2019). These studies have elucidated the architecture of prototypical eukaryotic promoters (Lenhard et al., 2012; Haberle and Stark, 2018). However, many unicellular eukaryotic organisms, like those belonging to the phylum Apicomplexa, differ extensively from model systems (Baldauf, 2003) and therefore demand tailored analyses of transcription initiation and regulation.

Apicomplexa include the causative agents of widespread human diseases, such as *Toxoplasma gondii*, *Plasmodium* spp., and *Cryptosporidium* spp. These parasites have complex life cycles that typically involve several developmental stages underpinned by distinct transcriptional programs. This complexity is illustrated by *Toxoplasma*, which can infect and replicate within any nucleated avian or mammalian cell, establishing both acute and chronic stages in intermediate hosts and undergoing sexual recombination within the definitive feline hosts. Asexual replication in intermediate hosts, like humans, is characterized by rapidly-dividing tachyzoites (Tz), which convert into the slow-replicating bradyzoites (Bz) that establish chronic infections lasting for the life of the host (Dubey, 2016). Transcriptome analyses using microarray and RNA sequencing (RNA-seq) methods have highlighted coordinate changes in the expression levels of numerous mRNAs as *Toxoplasma* transitions between intra- and extracellular environments (Hassan et al., 2017), converts between sexual and asexual stages (Fritz et al., 2012; Behnke et al., 2014; Farhat et al., 2020), and between acute (Tz) and chronic (Bz) stages (reviewed in Jeffers et al., 2018, also Garfoot et al., 2019; Ramakrishnan et al., 2019; Waldman et al., 2020; Xue et al., 2020).

Despite major progress in identifying the transcription factors that govern these transitions (e.g., Balaji et al., 2005; Farhat et al., 2020; Waldman et al., 2020), the lack of a comprehensive TSS map has limited advances in decrypting the interactions between *cis*- and *trans*-regulatory factors that converge at the promoters of regulated genes. The models of 8322 protein-coding genes in the *Toxoplasma* genome (ME49, ToxoDB v.45) were largely generated *via* computational prediction of coding sequences (CDSs) on the basis of RNA-seq and pre-RNA-seq expression data. However, 5′ and 3′ untranslated regions (UTRs), including TSSs, have remained incompletely defined due to the lack of data specifically addressing transcript boundaries. While standard RNA-seq is a powerful approach for quantifying gene expression, identifying splice isoforms, and discovering novel transcripts, it fails to accurately capture the 5′ end of transcripts, where information on TSSs and 5′ UTRs (also called 5′ leaders) is contained. This is because RNA-seq coverage is seldomly uniform, and typically diminishes toward the 5′ end of transcripts, owing to biases introduced during common sample preparation procedures (Wang et al., 2009; Levin et al., 2010). In particular, RNA-seq protocols do not select for 5′-intact mRNA, and instead often enrich mRNA *via* the polyadenylated tail, which favors 3′-end representation (Chao et al., 2019). In addition, the random fragmentation of mRNA reduces the efficiency of cDNA synthesis at transcript flanks, which contributes to the lack of sequencing coverage at 5′ ends (Wang et al., 2009). Indeed, available RNA-seq datasets in *Toxoplasma* are largely 3′-biased, and lack coverage of intact 5′ ends. This precludes the identification of 5′ termini at the resolution required to (i) correlate TSSs with sequence or chromatin elements that might influence gene expression, and to (ii) discern between and to accurately quantify distinct 5′ termini arising from alternative TSSs which can contribute to the functional or regulatory complexity of genes (Davuluri et al., 2008; de Klerk and ‘t Hoen, 2015).

Numerous assays have been developed for profiling transcription initiation genome-wide, all based on the capture and preferential sequencing of intact 5′ mRNA ends, called 5′ tags. In contrast to conventional RNA-seq, these 5′-end RNA-seq protocols provide high local coverage for precise TSS prediction, and enable the profiling of distinct TSSs that are linked to the same gene. A pioneering study published in 2010 provided the first such systematic assessment of transcription initiation in *Toxoplasma*, and suggested an absence of canonical eukaryotic core-promoter elements (Yamagishi et al., 2010). In *Plasmodium falciparum*, recent genomic mapping and profiling of TSSs throughout its intra-erythrocytic life cycle revealed a highly-complex and dynamic landscape of transcription initiation (Adjalley et al., 2016), suggesting a previously unknown diversity of transcripts with alternative 5′ termini. In *Toxoplasma*, the Tz-to-Bz stage conversion is accompanied by drastic changes in the mRNA expression levels of more than 2000 genes (Ramakrishnan et al., 2019; Waldman et al., 2020), but alternative stage-specific TSS usage has remained unexplored.

Here, we report an in-depth analysis of transcription initiation at *Toxoplasma* promoters in both acute (Tz) and chronic stages (Bz). Using state-of-the-art approaches for systematically characterizing mRNA 5′ ends (Batut and Gingeras, 2013; Adiconis et al., 2018), we generated a genome-wide map of transcription initiation at single-nucleotide resolution. We empirically defined dominant TSSs for the majority of *Toxoplasma* genes, revising most current gene models and providing an avenue for improved genome annotation through a comprehensive definition of 5′ leaders. Comparing TSS usage between Tz and Bz stages, we identified genes with alternative TSSs, some of which are regulated stage-dependently. Analysis of the nucleotide content and nucleosome occupancy around TSSs allowed us to examine the determinants for TSS choice, and outline features of *Toxoplasma* promoter architecture. Given the high complexity and pliability of *Toxoplasma* transcription, this report constitutes a highly-valuable resource for further investigations into the mechanisms directing TSS selection. Our study also provides a framework for functional genomics studies, specifically for targeted promoter manipulations using forward genetic approaches.

## Materials and Methods

### Parasites and Host Cells

*Toxoplasma* parasites were cultured at 37 °C in human foreskin fibroblasts (HFFs, ATCC SCRC-1041). Tz from the strains RH and ME49 were maintained at 5% CO_2_ in Dulbecco’s modified Eagle’s medium (DMEM; Gibco) supplemented with 3% or 10% heat-inactivated fetal bovine serum (IFS) and 10 µg/ml gentamicin (Thermo Fisher Scientific), referred to as standard medium. Bz from the strain ME49 were maintained at ambient CO_2_ in alkaline-stress medium, consisting of RPMI 1640 (Sigma), supplemented with 1% IFS and 10 µg/ml gentamicin, and buffered with 50 mM HEPES, adjusted to pH 8.1 with 10 N NaOH. HFFs that were destined for use in Bz experiments were maintained exclusively in DMEM supplemented with 10% IFS and 10 µg/ml gentamicin prior to infection.

### Bradyzoite Conversion and Harvest

ME49 parasites were grown in HFFs and standard medium, before changing to alkaline-stress medium at 24 h post-infection. At this time point, to remove residual standard medium, the infected HFF monolayer was washed once with alkaline-stress medium. Parasites were grown for an additional 48 h at 37°C at ambient CO_2_ to allow for stage conversion. The medium was then aspirated and HFF monolayers rinsed with PBS. Following addition of 3 ml PBS, the host-cell monolayer was detached by scraping, followed by the mechanical release of parasites by serially passing once through 27- and twice through 30-gauge needles before filtering through a polycarbonate filter with a 3-µm pore size (Whatman). The efficiency of stage conversion was estimated at 98%, as assessed by immunofluorescence staining and microscopy at the time of the Bz harvest. Guinea-pig anti-CDPK1, diluted 1:1000, provided a general parasite stain, while fluorescein-labeled dolichos biflorus agglutinin (DBA; Vector Laboratories), diluted 1:150, served as an early-Bz marker. DBA is a lectin that recognizes *N*-acetylgalactosamine on the Bz-specific cyst-wall protein CST1 (Tomita et al., 2013).

### Tachyzoite Harvest

RH Tz were allowed to egress naturally, while ME49 Tz were mechanically released. First, the medium was aspirated and HFF monolayers rinsed with PBS. Following the addition of 3 ml PBS, the host-cell monolayer was detached by scraping, followed by the mechanical release of parasites by passing through a 27-gauge needle. Egressed RH, and mechanically-released ME49 Tz were passed through a polycarbonate filter with a 3-µm pore size (Whatman).

### RNA Extraction and DNaseI Digest

Parasite suspensions were centrifuged for 5 min at 1000 × *g*, and washed once in PBS. Pellets were resuspended in TRIzol (Ambion) at about 1 ml per 5 × 10^6^ parasites, followed by vortexing, and incubating at room temperature for 5 min. Homogenized samples were flash-frozen in liquid N_2_ and intermittently stored at -80°C. Upon complete thawing at room temperature, samples were loaded onto MaXtract High Density tubes (Qiagen), and total RNA was extracted as per manufacturer’s instructions. RNA pellets were resuspended in nuclease-free water and concentration was determined using the Qubit RNA HS Assay Kit (Thermo Fisher). Genomic DNA contamination was removed from total RNA extracts using the TURBO DNA-free Kit (Invitrogen), following the manufacturer’s protocol (routine DNase treatment). Digest of DNA contaminants was confirmed by PCR on extracts before and after this procedure.

### Construction of RAMPAGE Libraries

RAMPAGE libraries were constructed from 5 µg of DNaseI-digested total RNA per biological replicate, largely following a previously published protocol (Batut and Gingeras, 2013; Batut et al., 2013) with the following modifications. (1) We extended the digest of 5′-monophosphate RNAs with 5′-phosphate-dependent exonuclease (Terminator Exonuclease; Lucigen) from 90 to 120 min since we observed high-level (> 50%) rRNA contamination in a pilot preparation using the shorter incubation time. (2) We used a universal template-switching oligo (TSO) instead of the barcoded ones, as published elsewhere (Adiconis et al., 2018). (3) We used a random 15-mer (RTP) with a modified tag sequence (universal TruSeq adapter; Illumina) for reverse transcription to allow for a separate index read, and a Read 2 using standard Illumina sequencing primers, as previously published (Adiconis et al., 2018). (4) In the final PCR, we used an extended forward primer (Fwd) with an optimized melting temperature, and (5) we used reverse primers (Rev) with 6-base indices for barcoding of individual libraries for multiplexed sequencing. (6) We used Q5 DNA polymerase (NEB) in the final PCR, and we first determined the optimal cycle number in small-scale reactions to prevent over-amplification in the subsequent bulk PCR reaction. (7) We used a different custom sequencing primer for Read 1 (R1), as previously published (Adiconis et al., 2018).

**Table:** 

**Designation**	**Sequence (5′ – 3′)**	
TSO	TAGTCGAACTGAAGGTCTCCAGCArGrGrG
RTP	TAGTCGAACGAAGGTCTCCCGTGTGCTCTTCCGATCT(N)15
Fwd	AATGATACGGCGACCACCGAGATCTACACTAGTCGAACTGAAGGTCTCCAG
Rev	CAAGCAGAAGACGGCATACGAGAT[index]GTGACTGGAGTTCAGACGTGTGCTCTTCCGATCT [index] = CTTGTA, GCCAAT, AGTTCC, TAGCTT, TTAGGC, ATCACG
R1	TACACTAGTCGAACTGAAGGTCTCCAGCAGGG

### Sequencing of RAMPAGE Libraries

Paired-end sequencing was performed with 75-nt reads, and a 6-nt index read. In a pilot, RH replicate libraries were sequenced on a MiSeq (Illumina), which revealed an ~85% G-nucleotide bias at the first position of Read 1, complicating the use of a NextSeq platform (Illumina) for sequencing of the full set of six RAMPAGE libraries. Read mapping to the ME49 genome assembly (v.45) revealed that ~85% of reads started with one non-encoded G, and ~10% of reads started with two non-encoded Gs, which were introduced in the non-templated addition of C nucleotides during reverse transcription. We therefore chose to employ an initial five chemistry-only (dark) cycles to enable sequencing on a NextSeq. 5′-tag positions were then shifted 5′ by 4 nt, which should match the position of the 5′-most nucleotide for ~85% of the original mRNA molecules, assuming all six libraries were structured similarly. This is expected to result in a minor loss of positional resolution. Analyzing the nucleotide composition around predicted TSSs revealed the canonical pyrimidine-purine dinucleotide at the -1 and 0 positions, which further validates the positional correction.

### Processing RAMPAGE Sequencing Data

Low-quality reads, and reads aligning to rRNA were filtered using TagDust2 v.2.33 (Lassmann, 2015) and a curated reference FASTA file containing all rRNA sequences annotated in the ME49 genome assembly (v.45), and the following settings:

-t 4 -dust 97 -fe 3 -1 R:N

Filtered reads were mapped onto the ME49 genome assembly (v.45) using STAR v.2.7.1a (Dobin et al., 2013) with the following settings:

–runMode alignReads –sjdbFileChrStartEnd [ … ] –alignIntronMax 6000 –alignSJoverhangMin 8 –alignSJDBoverhangMin 1 –readMatesLengthsIn NotEqual –alignMatesGapMax 1000 –outFilterMultimapNmax 1 –outFilterMismatchNoverLmax 0.04 –outSAMprimaryFlag AllBestScore –clip5pNbases 0 15 –outSAMtype BAM SortedByCoordinate –outSAMorder Paired

PCR duplicates were flagged within the BAM file using STAR v.2.7.1a with the following settings:

–inputBAMfile [ … ] –bamRemoveDuplicatesType UniqueIdentical –runMode inputAlignmentsFromBAM –bamRemoveDuplicatesMate2basesN 15

Flagged duplicates were then removed with SAMtools v.1.10 (Li et al., 2009) using:

samtools view -b -F 0x400

BedGraph files of 5′ tags were then generated from BAM files using STAR v.2.7.1a:

–inputBAMfile [ … ] –runMode inputAlignmentsFromBAM –outWigType bedGraph read1_5p –outWigNorm None

Positions of 5′ tags in the BedGraph files were shifted 5′ by 4 nt, and absolute values were normalized by dividing counts by the total number of counts per sample, and multiplying by 1000000 to generate counts per million (CPM).

### Data Analysis

Analyses were performed in Python or R, and plots generated in R (http://www.R-project.org) unless otherwise noted.

### Defining Gene-Association Windows

Strand-specific 5′-tag-count-to-gene–association windows were defined from -3500 nt to +200 nt relative to the start codons of the 8322 protein-coding genes (ME49 v.45). Upstream window sizes were shortened by the stop codon of an upstream gene on the same strand, or by contig boundaries.

### Density of 5′ Tags Across Gene-Association Windows

This analysis was restricted to (i) genes with unshortened gene-association windows, and (ii) genes with cumulative 5′-tag counts above the 1st percentile of the respective dataset, arriving at the sample sizes indicated in the graphs. The 5′-tag count at each position within individual gene-association windows was divided by the cumulative 5′-tag count for the respective window. Density values from biological replicates were then averaged for each position, followed by averaging densities for each position across all gene-association windows to generate 5′-tag counts as percent of cumulative 5′-tag counts. A scatter plot with a trendline and 95% confidence interval was generated in R using the ggplot2 package with the geom_smooth argument set to: method = “gam”, formula = y ~ s(x, bs = “cs”). The empirical cumulative density plot was also generated with these values.

### Defining *Toxoplasma* TSSs

TSSs were identified separately for each biological replicate as the position with the highest 5′-tag count within a gene-association window. Replicate TSSs were flagged as reproducible if they were within 40 nt, or as non-reproducible if they were farther than 40 nt apart. For reproducible TSSs, the rounded geometric center between the two TSSs was used for plotting and subsequent analyses, and are also available in a table compilation (**Data S1**). Genes with a TSS determined in only one of the replicates were discarded. Scatterplots of reproducible and non-reproducible TSSs were generated in R, as were violin and box plots of reproducible TSSs.

### Comparing ME49 Bz and Tz TSSs

This comparison was restricted to TSSs that were reproducible at the nucleotide level between biological replicates of ME49 Bz and Tz. TSSs between both stages were then compared by their distance to annotated start codons, whereas TSSs farther than 40 nt apart were flagged as putative stage-specific alternative TSSs. Genes for which a TSS was only identified in one stage were flagged as unique. The set of unique Bz genes (*n* = 1017) was defined as “inactive” genes in Tz for the analyses pertaining to the nucleosome occupancy around inactive Bz TSSs.

### Comparison of TSS Predictions to Current Models

A Tz-biased approach was chosen to curate a list of TSSs from both Bz and Tz stages. TSS predictions were successively added, only if not already present in the list, starting with TSSs that were reproducible to the nucleotide position between all ME49 replicates (category I), followed by reproducible to the nucleotide position between ME49 Tz replicates (category II), reproducible to the nucleotide position between ME49 Bz replicates (category III), reproducible within 40 nt between ME49 Tz replicates (category IV), reproducible within 40 nt between ME49 Bz replicates (category V), and reproducible at the nucleotide position between two ME49 replicates (category VI). For categories IV and V, the geometric center between TSSs from biological replicates was used to define the position of the TSS prediction. Predictions are available in a table compilation (**Data S1**) and BED format (**Data S2**) for display in genome browsers. Predictions from this study and TSS annotations in the ME49 reference annotation (v.45) were evaluated by their distance to annotated start codons of their associated genes.

### Generation of DNA and RNA Sequence Logos

Sequence logos were generated using the WebLogo v.3.7.5 command line tool (Crooks et al., 2004).

### RNA-Seq and Differential Expression Datasets

Data from conventional RNA-seq were previously published and used here as follows. For correlation analyses between RNA-seq TPM and RAMPAGE cumulative 5′-tag CPM, unstranded RNA-seq data was used from ME49 Tz and alkaline-stress–induced Bz (Waldman et al., 2020). ME49 Bz to Tz differential expression data was derived from these two ME49 datasets as previously published (Waldman et al., 2020); briefly, differential expression in Bz compared to Tz (log_2_ scale) was determined using the DESeq2 R package v.1.21.16 (Love et al., 2014), with an adjusted *p*-value cutoff of 0.001. For all visualizations of RNA-seq coverage, stranded RNA-seq data from an engineered ME49 Shield-1–inducible BFD1-overexpression strain was used, with data corresponding to populations maintained in the presence (Bz) or absence (Tz) of Shield-1 as described and analyzed previously (Waldman et al., 2020).

### MNase-Seq Data and Analysis

Previously published MNase-seq data from *Toxoplasma* Pru Tz were retrieved (Farhat et al., 2020). Raw sequencing reads were adapter- and quality-trimmed using Trim Galore v.0.4.1 (github.com/FelixKrueger/TrimGalore) (-quality 20 –illumina -stringency 3 –paired –length 10). Processed reads were then mapped onto the ME49 reference assembly v.45 using bowtie2 v.2.4.1 (Langmead and Salzberg, 2012; Langmead et al., 2019) using an inter-mate distance of 100–200 bp to avoid dinucleosomes and other artifacts (–local -D 20 -R 3 -N 1 -L 20 -i S,1,0.50 –no-unal –no-mixed –no-discordant –phred33 -I 100 -X 200). SAM files were converted into BAM files, sorted and then used to generate a BedGraph coverage track using bamCoverage v.3.2.0 from the deepTools2 package (Ramírez et al., 2016), with a bin size of 1 bp, filtering for fragment sizes from 130 to 200 bp, and normalizing coverage to the size of the ME49 reference assembly (–binSize 1 –normalizeTo1x 65669794 –ignoreDuplicates –minFragmentLength 130 –maxFragmentLength 200). All MNase-seq visualizations represent mean coverages around indicated TSS subsets. Geometries of phased nucleosomal arrays were defined around the TSSs that matched at the nucleotide position between ME49 Tz replicates. For this, local maxima in nucleosome density were called in R to define the average center positions of nucleosomes relative to TSSs. A length of nucleosomal DNA of 147 bp was assumed to calculate the upstream and downstream edges of each nucleosome.

### Frequency of Poly(dA:dT) Tracts

Tracts of at least four consecutive deoxyadenosines or deoxythymidines were detected using the Fuzznuc command line tool from EMBOSS v.6.6.0.0 (Rice et al., 2000) (-pattern AAAA -complement Yes). The frequency of poly(dA:dT) tracts was plotted within ±1000-bp windows centered around the TSSs that matched at the nucleotide level between ME49 Tz replicates.

### Sense and Antisense 5′-Tag Densities at TSSs

ME49 Tz sense and antisense 5′-tag counts were normalized to the sense maximum within ±1000-bp windows around TSSs that replicated at the nucleotide position between biological replicates of ME49 Tz. These sense and antisense 5′-tag densities were then averaged across all sense or antisense windows. The mean densities from both biological replicates were then averaged, and plotted as a graph with no binning. The values for heatmaps were scaled differently to aid visualization. Here, replicate-averaged sense and antisense 5′-tag counts were normalized separately to the respective window maximum, averaged over 20-nt bins diverging from a 21-nt bin centered around TSSs. Local minima and maxima in 5′-tag density were determined on data that was smoothed using a 20-nt rolling average. Minima and maxima were superimposed on the schematic representation of average nucleosome positions, and used to derive a cutoff distance for bidirectionally-paired TSSs.

### Detection of Bidirectionally-Paired Genes

Genes were sorted by the position of their start codons along chromosomes and contigs, followed by the pairing of adjacent gene pairs with opposite strandedness. Inter-TSS distances were calculated relative to the TSS of the upstream counterpart of each pair—upstream, as defined by the position of its start codon. Therefore, negative distances correspond to diverging TSSs, while positive values correspond to converging TSSs, which could result in overlapping transcripts. Distances were used for a density plot (bandwidth = 20) and a histogram (binwidth = 20 bp). A local minimum in antisense 5′-tag density demarcating the upstream border of the antisense cluster on the opposite pole of the NDR relative to the sense TSS was determined at -293 bp as described above. Diverging TSSs separated by 293 bp or less were considered to be bidirectionally-paired.

### Identification of a Novel Promoter Motif

Enrichment for the putative binding motif of BFD1 was previously determined (Waldman et al., 2020). A composite list of TSSs from Bz and Tz stages was curated based on differential expression analysis, evaluated on the basis of previously published stage-specific RNA-seq data (Waldman et al., 2020): (i) for genes upregulated in Bz (≥ log_2_-fold change of 2, *p*adj ≤ 0.001; *n* = 556), TSSs were selected from nucleotide-matching Bz replicates as determined above, and (ii) for genes downregulated in Bz or unregulated (*n* = 5412), TSSs were selected from nucleotide-matching Tz replicates as determined above. Motif analysis using MEME v.5.1.1 (Bailey and Elkan, 1994) was performed using sequences from -200 to 0 bp relative to TSSs (TSS windows; -dna -mod anr -nmotifs 3 -minw 5 -maxw 14 -objfun classic -revcomp -markov_order 0). MEME motif occurrences in the ME49 reference assembly (v.45) were identified using FIMO v.5.1.1 (Grant et al., 2011) with no strand-selectivity, and a *p*-value threshold of < 0.0001. A 40-bp rolling average of the frequencies of respective motifs and previously determined BFD1 CUT&RUN peaks (Waldman et al., 2020) in percent was calculated and plotted as a function of the distance to the TSSs of the subsets defined above.

### Interspecies 5′-Leader Length Comparison

Data for *Homo sapiens*, *Drosophila melanogaster*, *Danio rerio*, and *Arabidopsis thaliana* were compiled from a previously published meta-analysis of RefSeq data on 5′-leader lengths (Leppek et al., 2018). For plotting and calculating sample sizes and median lengths, all 5′ leaders shorter than two nucleotides were discarded (*Homo sapiens*, *n* = 44100; *Drosophila melanogaster*, *n* = 29862; *Danio rerio*, *n* = 14364; and *Arabidopsis thaliana, n =* 42704). *Toxoplasma* ME49 Bz and Tz 5′ leaders were derived from the midpoint of TSSs that replicated within 40 nt between biological replicates as described above. 5′-leader lengths were then calculated from the distance between TSSs and start codons—ignoring the possibility for introns that may occur within the defined 5′-untranslated regions. For plotting and calculating the sample sizes (Bz: 5837; Tz: 5612) and median lengths (Bz: 837 nt; Tz: 792 nt), all 5′ leaders shorter than two nucleotides were discarded. For defining TSSs and 5′ leaders in *P. falciparum* 3D7, we retrieved previously published CAGE data from the Gene Expression Omnibus data repository [GSE68982 (Adjalley et al., 2016)]. We processed BedGraph files containing 5′-tag counts for the 3D7 reference genome (version 11) for both replicates of all six investigated intra-erythrocytic time points. Gene-association windows were defined from -3500 to +200 nt around the start codons of all protein-coding genes in the 3D7 annotation. Upstream window sizes were shortened by the stop codon of an upstream gene on the same strand, or by contig boundaries. 5′-tag counts were associated with specific positions in gene-association windows for each sample. 5′-tag-count maxima (TSSs) were then detected in each of the 12 samples, discarding genes that had no maximum, or multiple maxima with identical 5′-tag counts. For each time point, replicate data was used to define TSSs that matched within 40 nt, and the midpoint between these TSSs was defined as the consensus TSS. A consolidated list of 3D7 TSSs was then generated *via* random selection of one TSS per gene from all samples. 5′-leader lengths were calculated from the distance between TSSs and start codons—ignoring the possibility for introns that may occur within the defined 5′-untranslated regions. For plotting and calculating the sample size (3763) and median length (431 nt), all 5′ leaders shorter than two nucleotides were discarded.

### Calculating Sizes of Exons, Introns, and Intergenic Spacers

A previously published python script [gtfstats.py (Francis and Wörheide, 2017)] was used to determine the length of each exonic, intronic, and intergenic feature in the genome annotation (GFF) files for *Toxoplasma* (TgME49 v.45) and *Homo sapiens* (GRCh38). This resulted in the following sample sizes for *Toxoplasma* vs. *Homo sapiens*: introns (40103 vs. 1716073), exons (49023 vs. 1716073), and intergenic spaces (7646 vs. 33985).

### uAUG and uORF Analysis

This analysis was performed on unspliced full-length 5′-leader predictions as defined above. We defined uAUGs as any AUG triplet upstream of the start codon of the CDS, and uORFs as any open reading frame with a minimal length of nine nucleotides, whose initiating codon lies within the 5′ leader. In the event of multiple initiation sites for a given stop codon, we selected the most distal in‐frame uAUG as the uORF start. Out-of-frame overlapping uORFs (category B) were only detected down to the first codon that overlapped with the main ORF. Expected values were determined from reshuffled datasets, and are the average of 15 different simulations. These reshuffled datasets were generated using the Python module ushuffle (Jiang et al., 2008), which reshuffled the sequence of each 5′ leader, maintaining the same length and dinucleotide composition.

## Results

### Generation and Sequencing of 5′-Intact cDNA Libraries From *Toxoplasma*

**Figure 1:**
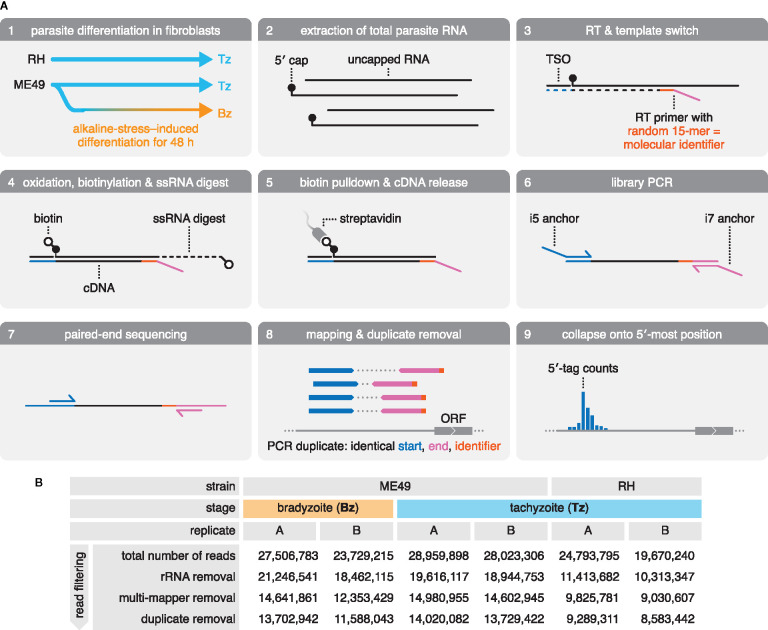
Generation and sequencing of 5′-intact cDNA libraries from total RNA extracts of multiple *Toxoplasma* strains and life-cycle stages. **(A)** Preparation and sequencing of 5′-intact cDNA (RAMPAGE) libraries. Total RNA was extracted from RH and ME49 Tz, as well as ME49 Bz that were differentiated by culture in alkaline-pH medium. Sequencing adapters were introduced during reverse transcription (RT). Here, an RT primer anneals randomly to RNA transcripts, introducing the Read 2 sequencing adapter (magenta), and a template-switching oligo (TSO) introduces the Read 1 sequencing adapter (blue) at the 5′ end of the RNA transcript. 5′-intact cDNA was enriched *via* a cap-trapping strategy, in which riboses with free 2′- and 3′-hydroxyl groups are oxidized and biotinylated, and single-stranded portions of RNA (ssRNA) are digested by RNase I. This retains biotin only at the cap-structure of 5′-intact RNA transcripts, which was used in a streptavidin pulldown to enrich for the 5′-intact cDNA within the RNA-cDNA heteroduplex. Libraries were amplified by PCR, introducing the i5 and i7 anchor sequences for Illumina flow cells. Following size selection, libraries were paired-end sequenced. Reads were mapped onto the ME49 genome assembly. Duplicate reads were identified by their identical alignment positions; however, over-collapsing was prevented by exploiting the fact that the RT primer often primes with mismatches, providing a pseudo-random molecular identifier. Deduplicated and filtered reads were then collapsed onto the first nucleotide of Read 1 to generate 5′-tag counts across the genome. ORF, open reading frame. **(B)** Summary of sequencing of RAMPAGE libraries and read filtering. rRNA, ribosomal RNA.

We adapted the RAMPAGE protocol (Batut and Gingeras, 2013) to systematically characterize mRNA 5′ ends in *Toxoplasma*, and to generate a genome-wide map of transcription initiation at single-nucleotide resolution. To capture strain- and life-cycle–dependent differences in transcription initiation, we constructed libraries from total RNA extracts of two canonical strains: the type I RH strain commonly used in cell culture and the type II ME49 strain frequently used in animal studies (**Figure 1A**, panels 1–2). Both strains were examined as acute-stage Tz, and ME49 was additionally analyzed as chronic-stage Bz, induced by culture under alkaline-pH stress.

The RAMPAGE protocol preserves the mRNA polarity in the resulting cDNA library by the directed introduction of Illumina-sequencing adapters for Read 1 at the 5′ end *via* a template switching oligo (TSO), and for Read 2 toward the 3′ end of the mRNA transcript *via* a randomly-priming reverse-transcription (RT) primer (**Figure 1A**, panel 3****). RAMPAGE employs two approaches to enrich for 5′-intact mRNA based on the presence of the 5′-m7G cap structure (cap): by (1) introducing the 5′-sequencing adapter *via* template switching, which preferentially occurs at the cap (Wulf et al., 2019), and by (2) biotinylation of the cap, followed by capture on streptavidin-coated beads (**Figure 1A**, panels 3–5****). Upon library amplification and paired-end sequencing, reads from both replicates of all three samples were mapped to the ME49 genome assembly (ToxoDB, version 45), and reads originating from ribosomal RNA (rRNA) contamination were removed (**Figure 1A**, panels 6–8;**** **Figure 1B**). Reads originating from PCR duplicates were removed to improve the accuracy of RNA quantification (**Figure 1A**, panel 8;**** **Figure 1B**). Upon removing rRNA and PCR-duplicated reads, libraries retained 8.5–14 million uniquely-mapping reads (**Figure 1B**). These reads were collapsed onto the first nucleotide of Read 1 to provide a frequency distribution of 5′ tags across the ME49 genome assembly—an approximation of transcription initiation activity (**Figure 1A**, panel 9;**** data available from GEO, accession number GSE159515).

### 5′-Tag Counts Are Correlated With Levels of Polyadenylation and Gene Expression

**Figure 2:**
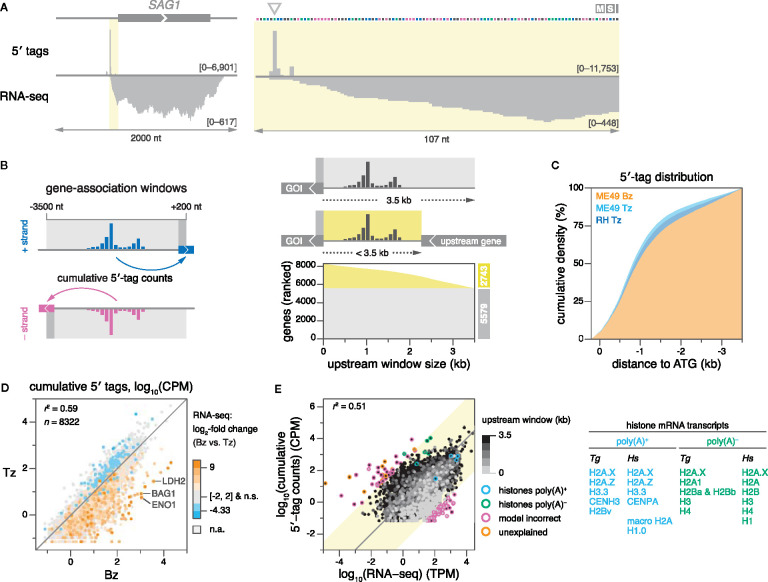
5′-tag counts correlate with polyadenylation and gene expression measured by RNA-seq. **(A)** 5′-tag and RNA-seq counts (ME49 Tz) on the (+) strand, upstream of the *SAG1* ORF (left), and zoomed in (right). Arrow indicates the major TSS previously determined (Burg et al., 1988). Data ranges indicated in counts per million (CPM) for 5′-tag data, and in transcripts per million (TPM) for RNA-seq data. **(B)** Strategy for associating 5′ tags to ME49 gene models. Strand-specific gene-association windows were defined around the start codons of 8322 protein-coding genes (left). The sum of all 5′-tag counts within a window constitutes a gene’s cumulative 5′-tag count. Where applicable, upstream windows were shortened by the stop codon of an upstream gene on the same strand, or by contig boundaries (right). The graph shows the upstream window size of genes in ranked order. **(C)** Empirical cumulative density plots of gene-level–normalized 5′-tag counts from proximal to distal positions within windows. GOI, gene of interest. **(D)** Correlation of cumulative 5′-tag counts between ME49 Bz and Tz. Genes are colored according to differential expression in Bz compared to Tz (log_2_ scale). Several canonical Bz genes are indicated. n.s., not significant. n.a., not applicable, i.e., genes below the coverage threshold required to calculate statistical significance. **(E)** Comparison of ME49 Tz cumulative 5′-tag counts (replicate-averaged) and Tz RNA-seq for relative quantification of gene expression (left). For clarity, genes with zero counts in either dataset are omitted. Genes are gray-shaded according to the size of their upstream gene-association window. RNA-seq libraries were generated using oligo(dT) priming for mRNA enrichment *via* poly(A) tails. Histone genes are highlighted according to the poly(A) status of their transcripts. Genes beyond three standard deviations (yellow background) are highlighted according to the accuracy of their model. Table of *Toxoplasma* (*Tg*) histone genes paired with their human (*Hs*) orthologues, grouped by the poly(A) state of their mRNA (right) (Dávila López and Samuelsson, 2008). The mRNA of H2A.X has both poly(A) and poly(A)-independent sequence motifs.

As a first assessment of the quality and shape of our data, we examined 5′-tag distributions at the well-studied promoter of the *SAG1* gene, and found that 5′ tags indeed peaked at the previously identified major TSS (Burg et al., 1988) (**Figure 2A**). To systematically associate 5′ tags with ME49 gene models, we defined strand-specific gene-association windows from -3500 to +200 nt relative to the start codons of all 8322 protein-coding genes in the reference annotation (version 45) (**Figure 2B**, left****). Windows extended 200 nt into annotated open reading frames (ORFs) to enable the capture of 5′ tags for genes with erroneously-annotated start codons. The upstream distance of 3500 nt was chosen to maximize the number of genes for which the full distribution of 5′ tags could be captured, while minimizing the risk of erroneously capturing pervasive transcription from neighboring genes. As a point of reference, we used annotations from the *Toxoplasma* genome database (ToxoDB.org), and determined that the upstream window size of 3500 nt captures nearly all (98.7%) current TSS annotations (**Figure S1A**). Where applicable, upstream windows were shortened to terminate at the boundaries of chromosomes or contigs, or at stop codons of upstream ORFs with matching strandedness (**Figure 2B**, right****). We found that 5′-tag density is normally-distributed across gene-association windows, with the mode of the distribution at ~700 nt upstream of the CDS and 50% of tags collected within ~900 nt proximal to the CDS (**Figure 2C**; **S1B**). These metrics suggest that the selected gene-association windows capture the bulk of gene-associated transcriptional activity in these samples.

As an approximation for transcript abundance, we integrated all 5′ tags within individual gene-association windows to generate cumulative 5′-tag counts for each gene (**Figure 2B**, left****). These values were highly-reproducible between the two biological replicates of each sample (**Figure S1C**). We then compared our measurements to expression data derived from conventional RNA-seq (Waldman et al., 2020), which served as an orthogonally-sourced metric for transcript abundance. Indeed, cumulative 5′-tag counts of replicate-averaged ME49 Bz and Tz samples recapitulated differential expression as evaluated by RNA-seq (**Figure 2D**). Transcript abundances assessed *via* conventional and 5′-end RNA sequencing (RAMPAGE) were positively correlated (*r* ^2^ = 0.51–0.55; **Figure 2E**; **S1D**), within the range reported by previous studies (Batut et al., 2013; Kawaji et al., 2014; Bhardwaj et al., 2019). Using the ME49 Tz datasets as an example, we investigated the discrepancy between gene expression measurements from RAMPAGE and RNA-seq. We analyzed genes known to differ in their polyadenylation to determine whether technical differences in transcript enrichment might underlie the discrepancy between the two methods, i.e., selecting mRNA *via* the 3′-polyadenylated (poly[A]) tail or the 5′-cap structure in RNA-seq or RAMPAGE, respectively. Indeed, we found that histone variants lacking poly(A) tails were underrepresented by RNA-seq, while those with poly(A) tails (López and Samuelsson, 2008; Marzluff et al., 2008) were quantified comparably, consistent with the generation of these RNA-seq libraries *via* oligo(dT) priming (**Figure 2E**). Furthermore, we observed that transcripts with shorter gene-association windows were more likely to have their gene expression underestimated by RAMPAGE compared to RNA-seq, which upon manual inspection of some examples we attributed to one or more of the following reasons: (i) a lack of sequence assembly upstream of the CDS, (ii) erroneous gene models of upstream genes, or (iii) an overall diminished capture of background pervasive transcription. We used both strand-specific RNA-seq (Waldman et al., 2020) and 5′-tag data to manually inspect outliers further than three standard deviations from the mean of the distribution. We found that the majority (91 of 102) is explained by inaccurate gene models or incomplete genome assembly [e.g., erroneously-annotated start codons; entirely spurious hypothetical gene models; transcripts split into multiple genes; erroneous annotation of introns, 5′ leader, and 3′ trailer sequences (**Figures S2A–H** for examples)], which cause erroneous transcript quantification by RNA-seq and/or cumulative 5′-tag counts (**Figure 2E**; **Table S1** for gene IDs). Finally, we investigated genes that had no coverage by conventional RNA-seq (*n* = 144), RAMPAGE (*n* = 224), or either technique (*n* = 27; **Table S2**). Manual inspection of some of these loci suggested that many genes with no or low coverage by either method may not be expressed in ME49 Tz, or their expression levels may be below the limit of detection. In addition, we noticed that genes not found by conventional RNA-seq were enriched for hypothetical proteins (72% vs. 51% among all genes) with generally shorter transcript lengths (973 nt vs. 3308 nt among all hypothetical transcripts), which could be indicative of spurious gene models that were predicted exclusively on the basis of short ORFs. Among the genes that were well-represented by conventional RNA-seq but lacked coverage by RAMPAGE, we frequently found inaccurate gene models, such as erroneous start codons or unresolved complex loci that prevented TSS capture, similar to the examples provided (**Figures S2A–H**).

In summary, our definition of gene-association windows provides a robust approach for the association of 5′-tag counts with gene models by demonstrating (i) the ORF-proximal accumulation of 5′-tag counts within gene-association windows, (ii) the reproducibility of cumulative 5′-tag counts, and (iii) the correlation of cumulative 5′-tag counts with conventional RNA-seq–derived transcript counts.

### Defining *Toxoplasma* Transcription Start Sites and 5′ Leaders

**Figure 3:**
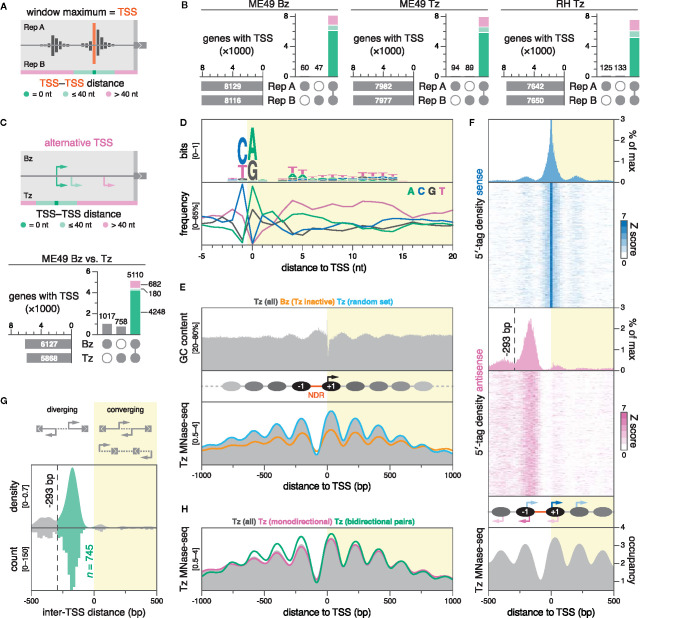
DNA sequence and nucleosome positioning are strong determinants of transcription initiation in *Toxoplasma*. **(A)** Strategy for TSS mapping. We defined the TSS as the nucleotide position with the maximal 5′-tag count within a gene-association window. High-confidence TSSs were selected by assessing their reproducibility across biological replicates. TSSs separated by 40 nt or less were considered to be within the same cluster. **(B)** UpSet plots showing the intersection of TSSs between biological replicates, colored by inter-TSS distance. Only TSSs that replicate at the exact nucleotide position are considered for subsequent plots. **(C)** Differential TSS usage between ME49 Bz and Tz was similarly evaluated, clustering TSSs separated by less than 40 nt. UpSet plot shows the intersection of nucleotide-matching Bz and Tz TSSs, colored by inter-TSS distance. **(D)** Nucleotide composition around ME49 Tz TSSs; *n* = 5866. **(E)** GC content around ME49 Tz TSSs (top) and nucleosome occupancy around different subsets of ME49 TSSs (bottom). Nucleosome occupancy was determined by MNase-seq on Tz of the *Toxoplasma* Pru strain (Farhat et al., 2020). Nucleosome occupancy in Tz around the set of unique Bz TSSs (orange; *n* = 1017) or a randomly-sampled subset of Tz TSSs of equal size (blue). **(F)** Periodicity of secondary and antisense transcription initiation corresponds to nucleosome positioning. Sense (blue) and antisense (magenta) 5′-tag counts displayed as densities or stacked values, above nucleosome occupancy (gray). 5′-tag counts were normalized to the sense maximum before collapsing into density plots. Stacked values represent 5′-tag counts normalized strand-specifically to the maximum intensity of each locus along 20-nt bins. As the proportion of sense 5′-tag counts amounts to ~95% at position 0, the histogram is cropped to visualize minor density patterns. The dotted line marks a local minimum in antisense 5′-tag density at -293 bp, which subsequently served as a distance threshold for defining diverging gene pairs as bidirectional. **(G)** Density plot (bandwidth = 20) and histogram (binwidth = 20 bp) of the distance between TSSs of adjacent gene pairs in ME49 Tz. Negative distances correspond to diverging (head-to-head) TSSs, while positive values correspond to converging TSSs, which could result in overlapping transcripts. Distances from -293 to 0 bp indicate putative bidirectional gene pairs (green). **(H)** Nucleosome occupancy around indicated subsets of ME49 Tz TSSs.

When assigning TSSs, we chose not to assess the spread of 5′ tags at individual promoters, and instead defined the TSS as the nucleotide position with the maximal 5′-tag count within a gene-association window (**Figure 3A**). As a measure of confidence for our predictions, we classified TSSs into three groups based on their reproducibility across biological replicates: (i) reproducible at the nucleotide position, (ii) reproducible within 40 nt, or (iii) not reproducible within 40 nt. The 40-nt cutoff was chosen semi-arbitrarily to allow for positional variability of TSSs within clusters while discarding TSSs that likely originate from transcriptional noise or spurious read mapping. Remarkably, most TSSs (67%–75%) were reproducible at the exact nucleotide position: 6127 (out of 8176) in ME49 Bz, 5868 (out of 8071) in ME49 Tz, and 5214 (out of 7775) in RH Tz (**Figure 3B**; **S3A**). An additional 9%–11% of TSSs were reproducible within 40 nt (713 in ME49 Bz, 729 in ME49 Tz, and 866 in RH Tz), half of which clustered within a 10-nt distance. The remaining 15%–18% of TSSs were not reproducible within 40 nt between biological replicates and were excluded from further analysis. Using TSSs replicable at the nucleotide position or within 40 nt, we determined that the distribution of TSS distances to annotated start codons was similar between ME49 Tz and Bz, as well as RH Tz (**Figure S3B**). For a number of genes (366 in ME49 Bz, 318 in ME49 Tz, 293 in RH Tz) TSSs were predicted downstream of annotated start codons; evaluation of these loci found these predictions to be indicative of erroneous gene models (**Data S1** for a comprehensive table).

Integrating TSSs from both ME49 Bz and Tz, we also curated a comprehensive set of predictions to serve as a resource for further studies and to amend current gene models. For the purpose of generating this set of predictions, we assumed that TSS usage does not change significantly between life-cycle stages (see analysis below), and thereby accumulated 7603 TSSs successively from six categories that may reflect the confidence of the prediction (**Figure S4A**). We categorized TSSs by their perfect agreement among all four ME49 samples (56%), the two Tz samples only (21%), or the two Bz samples only (13%). The remaining predictions used TSSs that agreed within a 40-nt window between Tz samples (5%) or Bz samples (3%), or at the same nucleotide between single Tz and Bz samples (2%). Collectively, we curated TSSs for 7603 genes, of which 5398 had predictions on ToxoDB (**Figure S4B**). Comparing predictions for these genes, we determined that 66% of our empirical predictions differed by more than 40 nt from those currently available on ToxoDB (**Figures S4B, C**), and we provide examples for how this data can improve current gene models (**Figure S4D**). Overall, our dataset provides empirically-determined TSSs for ~91% of *Toxoplasma* genes (**Figure S4E**; **Data S2** for BED file).

### Evidence for the Stage-Specific Use of Alternative TSSs in *Toxoplasma*

Alternative TSSs increase protein and regulatory diversity by giving rise to transcripts encoding protein isoforms with alternative N termini, or transcripts with distinct 5′ leaders that can entail differential translational regulation (Davuluri et al., 2008; de Klerk and ‘t Hoen, 2015). A recent study in *P. falciparum* identified numerous alternative TSSs, and profiled their differential activity during the parasite’s intraerythrocytic developmental cycle (Adjalley et al., 2016). In *Arabidopsis thaliana*, recent studies illustrated the systematic use of alternative TSSs as an adaptive mechanism in response to environmental stimuli (Ushijima et al., 2017; Kurihara et al., 2018). We wondered whether similar mechanisms may be present in *Toxoplasma*, and therefore explored the possibility of stage-specific alternative TSSs by comparing the high-confidence (nucleotide-matching) TSSs of ME49 Bz and ME49 Tz.

Analogously to our inter-replicate comparisons, we chose to employ a 40-nt distance cutoff for identifying TSS discrepancies between Bz and Tz (**Figure 3C**, top****). To increase specificity, we only considered TSSs that replicated at the exact nucleotide position in both replicates from a given stage. Comparing the resulting TSSs from ME49 Bz (6127) to Tz (5868), we identified 5110 genes for which TSS predictions exist in both stages (**Figure 3C**, bottom****). Identification of TSSs unique to ME49 Bz (1017) or Tz (758) correlated with their stage-dependent expression as measured by RNA-seq. Of the 5110 genes with predictions in both stages, 4428 genes had matching TSSs in both life-cycle stages, whereas 682 genes appeared to have alternative TSSs that were used in a stage-dependent manner (**Figure S5A**; **Data S3**). To evaluate the validity of this assignment, we manually inspected stage-specific stranded RNA-seq and 5′-tag data for 50 randomly-sampled loci from the 682 candidate genes (**Figure S5B**; **Table S3**). 24 of the sampled loci likely represent false positives for alternative TSS usage, with 15 explained by the TSS capture of pervasive transcription from neighboring differentially-expressed genes. However, we found 26 genes that clearly exhibited alternative TSSs, 16 of which showed a marked shift in stage-dependent usage, while 10 others had alternative TSSs that were used at similar levels stage-independently (**Figure S5C** for an example). Alternative TSS usage resulted in the gain or loss of upstream initiation triplets (uAUGs) and upstream ORFs (uORFs) in at least 17 of the 26 genes, which could conceivably affect translational regulation (**Table S3**). *Bona fide* stage-dependent alternative TSS usage was illustrated by *TGME49_200250*, where such a shift resulted in a 649-nt extension of the 5′ leader in Bz (**Figure S5D**). We note that while we have not systematically assessed differential activity of minor TSSs, we have observed such instances anecdotally, exemplified by *TGME49_262620*, where a minor TSS 317 nt upstream of the dominant TSS is specifically upregulated in Bz (**Figure S5E**). Collectively, we identified examples for alternative TSS usage, some of which are stage-dependent. Additional studies are needed to functionally characterize the different transcript isoforms that arise from these TSSs.

### DNA Sequence and Nucleosome Positioning Govern Transcription Initiation in *Toxoplasma*

Hallmarks of eukaryotic TSSs include distinct nucleotide motifs, so-called core promoter elements, as well as a nucleosome-depleted region (NDR) upstream of a phased nucleosomal array. Generating a genome-wide map of transcription initiation in *Toxoplasma* enabled us to assess the impact of these structural features on TSS choice. Centering individual genomic sequences only on TSSs that were reproducible at the nucleotide level between biological replicates, we found no remarkable difference in nucleotide composition around subsets specific to ME49 Bz, ME49 Tz, or RH Tz, and chose to present results using the set of TSSs from ME49 Tz in all subsequent plots (**Figure 3D**; **S6A**). We found a strong preference for a pyrimidine-purine dinucleotide at the -1 to 0 position relative to the TSS—a universal core promoter motif in both eukaryotic and prokaryotic organisms. A preference for thymidine at positions +3 to +25 corroborated the findings of a previous study that suggested a downstream thymidine cluster at positions +2 to +14 in *Toxoplasma* (Yamagishi et al., 2010). On a broader scale, we noticed a consistent bias against adenosine downstream of the TSS, congruent with a relative adenosine depletion in 5′ leaders, ORFs, as well as CDSs specifically (**Figure S6B**).

We also observed a sinusoidal oscillation in GC content, reminiscent of phased nucleosomal arrays (**Figure 3E**, top****). To relate this observation to data on nucleosome positioning, we reanalyzed publicly available MNase-sequencing (MNase-seq) data generated from Tz of the *Toxoplasma* Pru strain (Farhat et al., 2020). Surprisingly, we found that GC content anti-correlates with nucleosome density (**Figure 3E**, bottom****), which is in contrast to genome-wide studies in yeast and *in vitro* studies, where GC content is a strong predictor for nucleosome positioning (Lee et al., 2007; Peckham et al., 2007). GC-rich sequences inherently facilitate nucleosome formation while AT-rich sequences, specifically homopolymeric (dA:dT) tracts, disfavor the process (reviewed in Jansen and Verstrepen, 2011). We found that the frequency of poly(dA:dT) tracts indeed correlates with AT content and nucleosome density, suggesting that *Toxoplasma*-specific nucleosome-positioning factors may override purely biophysical DNA sequence propensities (**Figure S7**).

Just upstream of active *Toxoplasma* TSSs, we found a prominent NDR of ~91 bp, flanked by remarkably symmetric phased nucleosomal arrays with highly-positioned -1 and +1 nucleosomes (**Figure 3E**, bottom; **S8A**). The size of the average *Toxoplasma* NDR at active promoters is within the range described in yeast and metazoans (80–300 bp) (Raisner et al., 2005; Albert et al., 2007; Venters and Pugh, 2009). In yeast, the location of the TSS is just inside (~13 bp) the +1 nucleosome (Albert et al., 2007), whereas in metazoans, this nucleosome is positioned further downstream (~60 bp), leaving the TSS accessible within the NDR (Barski et al., 2007; Mavrich et al., 2008; Valouev et al., 2008). In *Toxoplasma*, we found that the TSS is located unusually deep into the +1 nucleosome, at ~41 bp from its upstream edge (**Figure S8A**). A nucleosome-internal TSS is compatible with a model described for yeast that suggests a role for the +1 nucleosome in facilitating transcription initiation (Albert et al., 2007).

Stage-specific data on transcription initiation also enabled us to compare nucleosome positioning at active and inactive *Toxoplasma* promoters. Studies in yeast and metazoans showed that nucleosome phasing and transcriptional activity are correlated and co-dependent (Tirosh and Barkai, 2008; Weiner et al., 2010; Oruba et al., 2020). Accordingly, inactive promoters are often characterized by minimal nucleosome phasing and the lack of an NDR (Mellor, 2005; Li et al., 2007; Lin et al., 2007; Jiang and Pugh, 2009). However, assessing Tz nucleosome positioning around the set of inactive Bz-specific TSSs (**Figure 3C**, bottom:**** unique set), we found that phasing, while reduced, was still detectable, and that the NDR was nearly as prominent as NDRs at randomly-sampled active promoters (**Figure 3E**, bottom****). The maintenance of nucleosome phasing, and the presence of a constitutive NDR in particular, could facilitate inducible activation by rendering Bz-specific promoters accessible for the binding of transcription factors during the Tz stage.

### Patterns of Pervasive Bidirectional Transcription at *Toxoplasma* TSSs

Although productive elongation may be unidirectional, generally, promoters are capable of initiating transcription bidirectionally (Neil et al., 2009; Xu et al., 2009). To assess the dynamics of bidirectional transcription within the nucleosomal context at *Toxoplasma* promoters, we projected averaged 5′-tag densities of both sense and antisense strands onto nucleosome density within ±1000-bp windows around ME49 Tz TSSs (**Figure 3F**; **S8A**). At these promoters, we found symmetrical patterns of bidirectional transcription initiation, clustered within nucleosomes. On the sense strand, the TSS accounts for the vast majority (~95%) of 5′ tags, yet secondary clusters peak at upstream (-198 nt) and downstream (+197 nt) locations, roughly corresponding to ±1 nucleosomal periodicity. Antisense 5′-tag density largely recapitulates these patterns: relative to the TSS, the major antisense cluster peaks at the opposite pole of the NDR (-169 nt), and is flanked by secondary clusters (-380 and +58 nt), again overlapping nucleosomes. We complemented averaged 5′-tag densities with heatmaps of 5′-tag counts for all ±1000-bp TSS regions, which showed that the observed patterns are indeed representative (**Figure 3F**). These patterns show that *Toxoplasma* promoters are inherently bidirectional, and that transcription initiates at regularly-spaced discrete loci within the nucleosomal array.

We wondered whether the symmetry in nucleosomal positioning, and incidence in antisense transcription at TSSs could also be indicative of bidirectionally-paired genes, i.e., non-overlapping (head-to-head) protein-coding genes that diverge from a common promoter. Bidirectional transcription of protein-coding genes is common in yeast, metazoans, and *P. falciparum* (Trinklein et al., 2004; Hermsen et al., 2008; Adjalley et al., 2016), and frequently allows for the co-regulation of functionally-related genes (Adachi and Lieber, 2002; Trinklein et al., 2004). To determine the proportion of the *Toxoplasma* genome that is organized into bidirectional gene pairs, we first defined such pairs as two adjacent genes whose coding sequences are located on opposite DNA strands, and with TSSs diverging from a shared NDR—a similarly strict definition was previously employed by genome-wide studies in yeast (Xu et al., 2009). We reasoned that antisense TSSs originating from the antisense 5′-tag cluster at the -1 nucleosome satisfy this definition. Consequently, we empirically derived a distance threshold between diverging TSSs to be considered as bidirectionally-paired by determining the upstream edge of the antisense 5′-tag cluster at the opposite pole of the NDR *via* a local minimum (-293 bp; **Figure 3F**). Using this threshold, we found 745 bidirectionally-arranged TSS pairs, corresponding to 1490 bidirectionally-paired genes, which represent a significant proportion (25%) of the genes considered in this analysis (*n* = 5868) (**Figure 3G**; **Data S4** for Gene IDs). In contrast to observations in *P. falciparum* (Adjalley et al., 2016), we found no evidence for an abundance of gene pairs with overlapping 5′ ends (**Figure 3G**, right****). Conversely, the striking enrichment among diverging gene pairs for bidirectionality is consistent with findings in *P. falciparum*, where ~23% of genes were arranged head-to-head, with TSS blocks separated by 400 bp or less (Adjalley et al., 2016). As expected, nucleosome phasing was more symmetrical at TSSs of bidirectionally-paired genes, given that both sides of the NDR lie downstream of a TSS (**Figure 3H**). Notably, correlating RNA-seq–derived data on expression and stage-dependent changes in expression between bidirectional gene pairs, we found no evidence for co-regulation (**Figure S8B**). Manual evaluation of a subset of putative bidirectional gene pairs on the basis of our stranded RNA-seq data, showed that the majority (~85%) of predictions indeed represent *bona fide* bidirectional pairs; however, our analysis also captured artefacts, caused by (i) the erroneous association of antisense transcription from highly-active upstream promoters of neighbouring genes, or (ii) by the pairing with spurious hypothetical gene models that appear to have been annotated on the basis of antisense transcription from the same promoter (**Figure S8C** for examples). We therefore note that the presented number of bidirectional gene pairs may be overestimated.

To summarize, we found pervasive bidirectional transcription at *Toxoplasma* promoters, that initiates within discrete clusters, localized deep into nucleosomes. This is consistent with a model described for yeast, in which the transcriptional machinery assembles in bidirectional configuration at internucleosomal spaces, and transcription initiation is facilitated by the respective +1 nucleosome (Albert et al., 2007). Similar to *P. falciparum*, we found that *Toxoplasma* organizes about a quarter of its protein-coding genome in bidirectional pairs. The lack of evidence for co-regulation of adjacent genes suggests mechanisms for the directional control of transcription.

### Discovery of a New Sequence Motif Using Nucleotide-Resolution TSS Data

**Figure 4:**
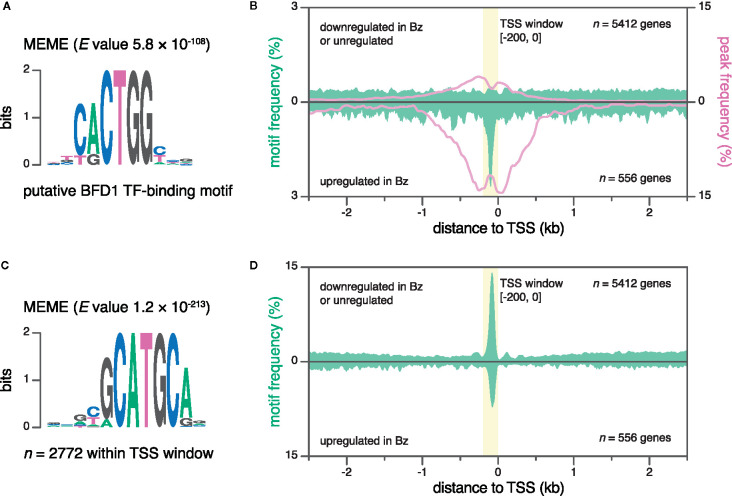
Discovery of new sequence motifs using base-pair-resolution transcription start site (TSS) data. **(A)** Putative binding motif of the transcription factor BFD1 (Waldman et al., 2020). **(B)** Frequencies for the putative BFD1 motif (green, left y-axis) and CUT&RUN peaks (magenta, right y-axis) around different TSS subsets. Top, ME49 Tz TSSs of genes that are either downregulated (≥ 2-fold change on a log_2_ scale) in ME49 Bz or not regulated, as evaluated by RNA-seq. Bottom, ME49 Bz TSSs of genes that are upregulated in ME49 Bz (≥ 2-fold change on a log_2_ scale). A TSS window (-200 to 0 bp; yellow) captures the peak in motif frequency at TSSs of genes upregulated in ME49 Bz. Data was smoothed using a 40-bp rolling average. **(C)** The MEME motif (gCATGCa) was significantly enriched within TSS windows combined from both ME49 Bz and Tz. **(D)** Mean gCATGCa-motif frequency around different TSS subsets, as in **(B)**.

Transcriptional regulation is mediated by the collective binding of transcription factors to *cis*-regulatory elements in promoter and enhancer DNA. At promoters, transcription-factor–binding sites are typically positioned at defined distances relative to the TSS (Lim et al., 2004; Vardhanabhuti et al., 2007). Therefore, nucleotide-resolution data on TSSs is key for both the prediction of regulatory motifs, as well as the association of experimental data on transcription factor binding and motifs with annotated genes. Recently, we identified the transcription factor BFD1, a master regulator of Bz differentiation, and determined its genomic binding sites and putative binding motif *via* CUT&RUN (Waldman et al., 2020) (**Figure 4A**). Here, we assessed the frequency of both the putative BFD1 binding motif and CUT&RUN peaks within 5-kb windows centered on the TSSs of genes upregulated in Bz, compared to those that were downregulated in Bz or expressed similarly across the two life-cycle stages (**Figure 4B**). The aggregate signals for both CUT&RUN peaks and binding-motif frequency were enriched within a narrow interval, peaking at ~98 bp upstream of the TSSs of genes upregulated in Bz. Therefore, like most *cis*-regulatory sequences, the BFD1 binding site resides in the NDR, where the binding of transcription factors is not obstructed by the presence of nucleosomes (Lee et al., 2004). To discover novel motifs, we defined an interval at -200 to 0 bp relative to the TSS, reasoning that additional motifs may be positioned similarly to those bound by BFD1. Using the MEME program for motif discovery (Bailey and Elkan, 1994), we identified significant enrichment of the gCATGCa motif within the TSS windows of all genes (**Figure 4C**). Specifically, this motif was present in 44% of TSS windows (2637 out of 6053), and most concentrated at ~82 nt upstream of TSSs, independent of gene regulation in Bz (**Figure 4D**). The gCATGCa sequence resembles two known motifs: the sporozoite-specific regulatory motif PfM24.1 (CATGCA) identified in *P. falciparum* (Young et al., 2008)—the putative binding motif of the *P. falciparum* ApiAP2 transcription factor PF14_0633 (YGCATGCP) (De Silva et al., 2008)—and the consensus binding sequence of plant-specific B3-domain–containing proteins (CATGCA; also known as RY element) (Suzuki et al., 1997). Collectively, our nucleotide-resolution data on TSSs enabled (i) the association of transcription-factor–binding data and a known binding motif to regulated genes, and (ii) the identification of a new putative *cis*-regulatory element. Similarly, future studies may use this data as a framework for motif discovery, and for assessing their regulatory potential.

### *Toxoplasma* 5′ Leaders Are Unusually Long and Lack Suppression of uAUGs and uORFs

5′ leaders serve as the entry point for the ribosome during cap-dependent translation, and can regulate gene expression at the post-transcriptional level by affecting translational efficiency and mRNA localization. Between individual genes, 5′-leader lengths can vary dramatically, ranging from a few, to thousands of nucleotides (Leppek et al., 2018). The median length of 5′ leaders in non-apicomplexan eukaryotes varies between approximately 53 nt in yeast and 218 nt in humans (Leppek et al., 2018); however, previous data have suggested that 5′ leaders are significantly longer in apicomplexans, including *Toxoplasma*, *P. falciparum*, and *Neospora caninum* (Yamagishi et al., 2012; Russell et al., 2013; Caro et al., 2014; Ramaprasad et al., 2015).

**Figure 5:**
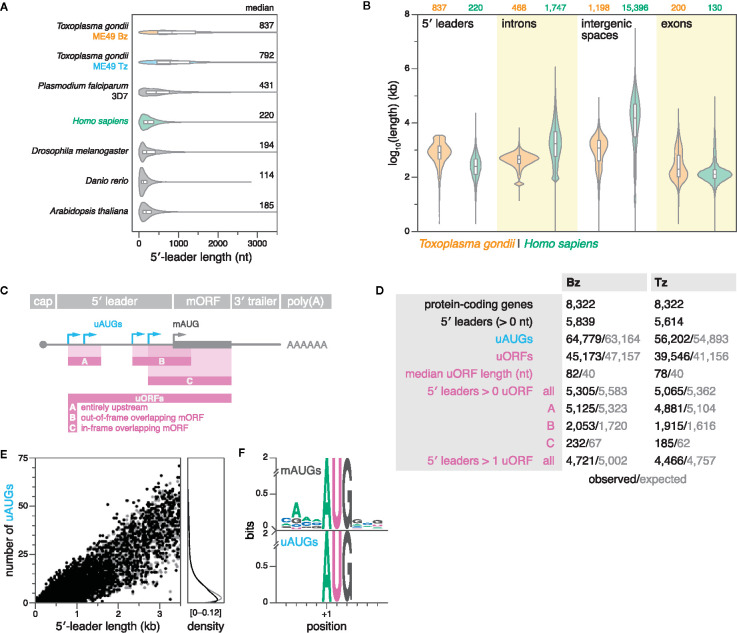
*Toxoplasma* 5′ leaders are unusually long and lack suppression of upstream initiation triplets (uAUGs) and upstream ORFs (uORFs). **(A)** Violin and box plots showing the distribution of 5′-leader lengths in various species. Outliers were removed for clarity. **(B)** Violin and box plots showing the length distributions of 5′ leaders (this study), introns, intergenic spaces, and exons in ME49 Bz and humans. Outliers are omitted for clarity. **(C)** Definitions of uAUGs and uORFs. uORFs have a minimum length requirement of nine nucleotides. In the case of multiple in-frame overlapping uORFs that share the same stop codon, only the longest was reported. **(D)** Summary statistics of uAUGs and uORFs in ME49 Bz and Tz. Simulated values represent the average of 15 different simulations, in which 5′-leader sequences were individually shuffled while maintaining dinucleotide frequency. Refer to previous panel for uORF categories. **(E)** Left, scatter plot showing the number of uAUGs per gene as a function of 5′-leader length in ME49 Tz. Right, corresponding density plot. **(F)** Sequence logos of weighted position-specific scoring matrices showing nucleotide conservation around mAUGs (top; *n* = 5614) and uAUGs of uORFs (bottom; *n* = 39546) in ME49 Tz.

Here, we comprehensively compared the length distribution of 5′ leaders between apicomplexans, and other model eukaryotes. First, we compiled data from a meta-analysis of RefSeq data on 5′-leader lengths from several model eukaryotes, including *Homo sapiens*, *Drosophila melanogaster*, *Danio rerio*, and *Arabidopsis thaliana* (Leppek et al., 2018). Secondly, we added 5′-leader data from (i) *Toxoplasma* as defined by TSSs that were predicted upstream of CDSs, and replicated within 40 nt in both biological replicates of ME49 Bz or Tz, and (ii) *P. falciparum* 3D7 as defined strictly analogously, using publicly-available 5′-end RNA-seq data (Adjalley et al., 2016) (see materials and methods for details). Indeed, the median 5′-leader lengths of both *Toxoplasma* (792–837 nt) and *P. falciparum* (431 nt) were substantially longer than those of the other analyzed eukaryotes (114–220 nt) (**Figure 5A**). These results suggest that extended 5′ leaders may be a conserved feature among diverse apicomplexan species. Remarkably, the expansion of 5′ leaders in *Toxoplasma* relative to other eukaryotes such as humans (median lengths of 792–837 vs. 220 nt) contrasts with its otherwise compact genome reflected in the reduced median lengths of introns (468 vs. 1747 bp) and intergenic sequences (1198 vs. 15396 bp) (**Figure 5B**).

A long region between the point of transcription initiation and the translation-initiation site in the mature mRNA provides a substrate for the evolution of mechanisms for post-transcriptional regulation. Various 5′-leader–localized linear and structural elements can modulate translation initiation (Wilkie et al., 2003; Jackson et al., 2010; Leppek et al., 2018). We therefore wondered whether the expansion of 5′ leaders may have been accompanied by a proliferation of mechanisms for post-transcriptional regulation. Specifically, we chose to analyze the occurrence of uAUGs and uORFs, which are common *cis*-regulatory elements in mammalian transcriptomes, often modulating the translational activity of downstream protein-coding ORFs by sequestering ribosomes (Morris and Geballe, 2000; Iacono et al., 2005). Here, we defined uAUGs as any AUG triplet upstream of the start codon of the CDS (main AUG, or mAUG), and uORFs as any open reading frame with a minimal length of nine nucleotides, whose initiating codon lies within the 5′ leader (**Figure 5C**). In our curated set of ME49 Bz and Tz 5′ leaders, we detected putative uORFs in ~90% of transcripts, surpassing the frequency observed in mammals (~50%) (**Figure 5D**; **Data S5** and **S6** for BED files) (Iacono et al., 2005).

As expected, the number of uAUGs and uORFs correlated with 5′-leader length (**Figure 5E**; **S9**). In a range of eukaryotes, genome-wide studies have previously demonstrated that there are significantly fewer AUG triplets in 5′ leaders than expected by chance, which suggested that uORFs tend to be depleted from 5′ leaders due to their deleterious effects (Rogozin et al., 2001; Lynch et al., 2005; Neafsey and Galagan, 2007). To assess whether the occurrence of uAUGs and uORFs in *Toxoplasma* differed from what would be expected by chance, we compared observations with expectations calculated from reshuffled sequences of identical length and dinucleotide composition (see materials and methods for details). 5′ leaders with 1–6 uAUGs or 1–5 uORFs were slightly less prevalent than expected; however, there was no consistent deviation between observed and expected occurrences of these features, suggesting reduced selection against them in *Toxoplasma* (**Figures 5D, E**; **S9**).

Sequences immediately surrounding an AUG triplet can dramatically affect the frequency of translation initiation (Kozak, 1978; Kozak, 1986). Favourable consensus sequences for translation initiation have been defined in various species including *Toxoplasma* (Seeber, 1997). Importantly, in contrast to the conserved sequence context around mAUGs, we found that the sequences surrounding uAUGs were random, suggesting that uAUGs typically represent weak start codons (**Figure 5F**). This is consistent with the findings of a recent study that assessed the translational potential of some sequence-based uORF predictions in *Toxoplasma via* ribosome profiling, suggesting that uORF translation is typically inefficient, and that both mRNA sequence and secondary structure context may regulate translation efficiency (Hassan et al., 2017).

Collectively, we found that *Toxoplasma* 5′ leaders are on average among the longest of any eukaryote studied thus far. The occurrence of uAUGs and uORFs is generally not suppressed, and we find that sequence context may provide one mechanism to privilege a particular AUG for translation initiation by the *Toxoplasma* ribosome.

## Discussion

The lack of defined *Toxoplasma* TSSs has impeded genomic efforts in this divergent eukaryote. Using 5′-end RNA-seq, we mapped transcription initiation activity at single-nucleotide resolution in acute and chronic stages of *Toxoplasma*, providing a much-needed resource for functional genomics studies. We empirically defined TSSs for ~91% of *Toxoplasma* genes, effectively revising 68% of current models and providing an avenue for improved genome annotation. We identify genes that putatively exhibit alternative TSSs, some of which appear to be differentially regulated in a stage-specific manner suggesting an additional transcript diversity that has not previously been appreciated. The canonical pyrimidine-purine dinucleotide motif at *Toxoplasma* TSSs is accompanied by sequence patterns such as a downstream thymidine cluster and a large-scale sinusoidal oscillation in GC content that appears anticorrelated with nucleosome occupancy. The *Toxoplasma* TSS lies unusually deep within nucleosomes, and is preceded by a prominent NDR that is at the center of a highly-symmetric phased nucleosomal array. Pervasive bidirectional transcription initiates with a defined pattern along the nucleosomal array. Demonstrating the utility of this data, we found that the putative binding motif of BFD1, a master regulator of Bz differentiation, resides at a specific distance from the TSSs of targeted genes, and we identified a novel motif with a similar positional arrangement at 44% of *Toxoplasma* promoters. Corroborating previous observations, we asserted that *Toxoplasma* 5′ leaders are among the longest of any eukaryote studied thus far. We found that 5′ leaders lack suppression of uAUGs and uORFs, and we determined that sequence context may privilege mAUGs for translation initiation by the *Toxoplasma* ribosome. Collectively, our work provides a framework to investigate the interactions between genomic sequences and regulatory factors governing the complex transcriptional program of this parasite.

The *Toxoplasma* reference annotation (ToxoDB v.45) was largely generated *via* computational CDS prediction on the basis of RNA-seq and pre-RNA-seq expression data. However, untranslated regions, including TSSs, have remained incompletely defined due to the lack of integration with specific data on transcript boundaries, which has complicated investigations into the molecular mechanisms of transcriptional control. A pioneering study from 2010 used oligo-capping (Suzuki and Sugano, 2003) for the enrichment of 5′-intact cDNA and provided the first systematic assessment of transcription initiation in *Toxoplasma* (Yamagishi et al., 2010); however, these data never informed transcript annotations, and substantially more accurate and specific approaches have since been developed (Adiconis et al., 2018). With RAMPAGE (Batut and Gingeras, 2013), we used state-of-the-art approaches for systematically characterizing mRNA 5′ ends, thereby generating a genome-wide map of transcription initiation at single-nucleotide resolution. We empirically define dominant TSSs for 91% of protein-coding genes, compared to 68% of reference annotations, of which we extensively revised 66%. Apart from missing or erroneous TSS annotations, we identified various other gene-model inaccuracies, and putatively spurious models of hypothetical genes, highlighted by discrepancies between conventional and 5′-end RNA-seq. Future integration of the two modalities of strand-specific RNA-seq data—conventional and 5′-end—may facilitate the automated generation of high-quality empirical genome annotations (Boley et al., 2014; Wang et al., 2019). The integration of these datasets will likely be key in the *de novo* annotation of non-coding RNAs in *Toxoplasma*. Generating similar datasets from other life-cycle stages of *Toxoplasma* will be important in assessing stage-specifically–expressed genes that could not be captured in the present study. In addition, integrating our stage-specific 5′-end data with emerging long-read RNA-seq data from published (Lee et al., 2020) and future studies will likely improve the association of (alternative) TSSs to annotated genes.

We found evidence for the stage-specific use of alternative TSSs in *Toxoplasma*. Alternative TSSs are common in eukaryotic genomes where they increase protein and regulatory diversity by giving rise to transcripts encoding protein isoforms with alternative N termini, or transcripts with distinct 5′ leaders that can impart differential translational regulation (Trinklein et al., 2003; Zhang et al., 2004; Davuluri et al., 2008; FANTOM Consortium and the RIKEN PMI and CLST (DGT) et al., 2014; Haberle et al., 2014; Ushijima et al., 2017; Kurihara et al., 2018). Numerous alternative TSSs are differentially used during progression of *P. falciparum* through its intra-erythrocytic life-cycle stages (Adjalley et al., 2016). We note that our analysis focused on the identification of stage-dependent shifts between dominant TSSs in *Toxoplasma*; however, we have not comprehensively identified minor TSSs, or assessed their differential activity. Such an example is illustrated by the gene *TGME49_262620* where appearance of a minor TSS 317 nt upstream of the dominant TSS suggests the existence of a transcript isoform with an expanded 5′ leader specifically in Bz. Shifts in dominant TSSs were observed at some loci, such as *TGME49_200250*, where alternative TSS usage results in a 649-nt extension of its 5′ leader in Bz. Experimental validation will be needed to assess the functional impact that these 5′-leader extensions may have. Since our RAMPAGE data reflects transcription initiation activity within a population of parasites at various stages of their cell cycle, it will also be interesting to investigate synchronized cultures of *Toxoplasma* to resolve any cell-cycle–dependent shifts between alternative TSSs.

Canonical eukaryotic core promoter elements are hardly used in *Toxoplasma* and related *P. falciparum* (Yamagishi et al., 2010; Adjalley et al., 2016). Transcription initiates at the universal pyrimidine-purine (YR) dinucleotide (-1, 0), which is thought to facilitate the transition of RNA polymerase II from transcription initiation to mRNA elongation (Winkelman et al., 2016). While the YR dinucleotide may be required for efficient transcription initiation at a given site, it lacks the selectivity to explain the tight distribution of *Toxoplasma* TSSs. We corroborated the presence of a *Toxoplasma*-specific downstream thymidine cluster (+3–25) (Yamagishi et al., 2010), which may facilitate positioning of the +1 nucleosome and the preinitiation complex. Similar to other eukaryotes, the *Toxoplasma* TSS and the +1 nucleosome have maintained a fixed distance, suggesting that their positions have not arisen independently. The metazoan TSS resides within the NDR, ~60 nt upstream of the +1 nucleosome (Barski et al., 2007; Mavrich et al., 2008; Valouev et al., 2008), whereas *S. cerevisiae* initiates transcription ~13 nt inside the +1 nucleosome (Albert et al., 2007). In *Toxoplasma*, transcription initiates even deeper into the +1 nucleosome, clustered at around ~41 nt inside the nucleosome’s upstream edge. Histone acetylation marks at the +1 nucleosome are thought to recruit and position the preinitiation complex at the TSS of *S. cerevisiae* (Jacobson et al., 2000; Matangkasombut et al., 2000; Hassan et al., 2002), and similar mechanisms may focalize transcription initiation in *Toxoplasma*.

At *Toxoplasma* promoters, we found that transcription initiates bidirectionally within discrete clusters that co-localize with the nucleosomes of a highly-symmetric phased nucleosomal array. Such patterns of pervasive divergent transcription at eukaryotic promoters are common (Jensen et al., 2013). However, the unusually-pronounced nucleosome phasing upstream of promoter-associated NDRs in *Toxoplasma* may structurally enhance the incidence of antisense transcription. Consistent with the presence of bidirectional activity and upstream nucleosome phasing, we found that *Toxoplasma* may organize up to a quarter of its protein-coding genome as bidirectional pairs that diverge from a common NDR. This configuration is similarly prevalent in *P. falciparum* (Adjalley et al., 2016). Manually evaluating a subset of putative bidirectional gene pairs on the basis of our stranded RNA-seq data, we found that ~15% were artefactual, caused by the erroneous TSS capture of antisense transcription from highly-active upstream promoters of neighbouring genes, or by the pairing with spurious hypothetical gene models from the ME49 reference annotation that may have been predicted based on antisense promoter transcription. We therefore note that the presented number of bidirectional gene pairs may be overestimated. Such loci are likely to be resolved with additional analyses, improved genome annotations, and particularly with the integration of data from stranded long-read 5′-end and conventional RNA-seq. In metazoans, bidirectional gene pairs are also frequent (Trinklein et al., 2004; Hermsen et al., 2008), and often indicative of co-regulated adjacent genes with shared *cis*-regulatory elements (Adachi and Lieber, 2002; Trinklein et al., 2004); however, we could not establish a co-regulatory relationship between bidirectionally-paired genes in *Toxoplasma*, suggesting the presence of separate regulatory elements that allow for the directional control of promoter activity (Bagchi and Iyer, 2016). Even in the absence of shared *cis*-regulatory elements, the proximity between TSSs of bidirectionally-paired genes must be considered in forward genetic approaches that hinge on targeted promoter manipulations, such as CRISPR interference and activation (Rosenbluh et al., 2017).

Nucleosome positioning in *Toxoplasma* does not correlate with canonical sequence determinants. In yeast and metazoans, GC content is a strong predictor of nucleosome positioning (Lee et al., 2007; Peckham et al., 2007), and NDRs are often enriched in poly(dA:dT) tracts, which inherently disfavor nucleosomes (Yuan et al., 2005). By contrast, we found that nucleosome occupancy at *Toxoplasma* promoters strictly follows oscillations in AT content and poly(dA:dT) frequency, suggesting that *Toxoplasma*-specific nucleosome positioning factors may override intrinsically unfavorable DNA sequences.

We found that nucleosome phasing at inactive Bz promoters is maintained during the Tz stage. The maintenance of nucleosome phasing, and the presence of a constitutive NDR in particular, is consistent with a model in which Bz promoters are structurally poised for activation already prior to stage conversion. This would be similar to observations at stimulus-inducible cell-type–specific promoters in mammals (Oruba et al., 2020), where the magnitude of transcription after activation has been shown to correlate with promoter nucleosome depletion (Scruggs et al., 2015). The transcription factors bound at these NDRs and the chromatin remodelling factors involved in maintaining nucleosome patterning at Bz promoters remain to be identified. However, we note the following caveats to this analysis, in that (i) we cannot exclude potential Bz contamination in the MNase-seq data, and (ii) by assessing the gross average of nucleosome positioning across all Bz-specific TSSs, we ignored the potential presence of heterogeneous promoter patterning between individual genes. With promoters mapped in the asexual stages, and with the advent of an inducible system for *Toxoplasma* stage conversion (Waldman et al., 2020), future work may study the dynamics of nucleosome positioning at stage-dependent *Toxoplasma* promoters in greater detail.

We found that the putative BFD1 transcription-factor-binding motif is positioned at a defined distance relative to the TSSs of genes upregulated in Bz, consistent with its role as a master regulator for Bz differentiation (Waldman et al., 2020). We also identified a novel gCATGCa motif present at similarly defined positions upstream of 44% of *Toxoplasma* TSSs. The remarkable prevalence of this motif suggests an eminent role in regulating transcription, but its cognate transcription factor remains unidentified. The positioning of the BFD1 and gCATGCa motifs is typical for *cis*-regulatory elements in that they are at defined distances relative to the TSS (Lim et al., 2004; Vardhanabhuti et al., 2007), and reside roughly at the center of promoter-associated NDRs, where they are accessible for the binding of transcription factors (Lee et al., 2004). The herein-defined high-resolution TSSs for most *Toxoplasma* genes will likely facilitate similar studies into the association between transcription-factor-binding data, motifs, and annotated genes.

Our data corroborates that 5′ leaders of both *Toxoplasma* and *P. falciparum* are on average among the longest of any eukaryote studied thus far, suggesting that extended 5′ leaders may be a conserved feature among diverse apicomplexan species. Since 5′ leaders are associated with mechanisms of post-transcriptional regulation (Wilkie et al., 2003; Sonenberg and Hinnebusch, 2009; Jackson et al., 2010), the elongation of these non-coding regions may have contributed to increased regulatory complexity. However, 5′ leaders also provide a substrate for the mutational origin of potentially deleterious uAUGs and uORFs. These features are prevalent and regulatory in a variety of eukaryotes, including *P. falciparum* (Amulic et al., 2009; Caro et al., 2014), and typically occur significantly less frequently than would be expected by chance (Rogozin et al., 2001; Lynch et al., 2005; Neafsey and Galagan, 2007). In *Toxoplasma*, however, we found an abundance of putative uAUGs and uORFs, and no evidence for purifying selection against these features. The overall lack of statistical deviation between observed and expected frequencies of uAUGs and uORFs suggests that these elements are often irrelevant. This conclusion is consistent with ribosome-profiling data which found that putative *Toxoplasma* uORFs are typically not translated (Hassan et al., 2017); however, a more comprehensive functional evaluation of the herein-defined uORFs on the basis of both ribosome-profiling and proteomics data is ultimately needed. Mechanistically, the weak sequence context for translation initiation that we and others found at uAUGs may enable the *Toxoplasma* ribosome to bypass (through ‘leaky scanning’) these elements, and instead initiate translation at the downstream mAUG (Kozak, 1986; Hassan et al., 2017), and mRNA secondary structure around mAUGs has also been proposed to define the sites of translation initiation in *Toxoplasma* (Hassan et al., 2017).

Our precise genome-wide mapping of TSSs constitutes a major advance toward deciphering the molecular basis of both transcriptional and translational control in *Toxoplasma*. These predictions will both benefit from and also inform future revisions of reference assemblies and annotations. This resource opens new avenues into the characterization of the mechanisms that direct TSS choice and regulate promoter activity, and will be essential for the development of functional genomics tools like CRISPR activation and interference in *Toxoplasma*.

## Data Availability Statement

The datasets presented in this study are accessible through online repositories. Raw RAMPAGE sequencing data, and processed BedGraph files of strand-specific 5′-collapsed read coverage have been deposited in NCBI's Gene Expression Omnibus (Edgar et al., 2002) and are accessible through GEO Series accession number GSE159515 (https://www.ncbi.nlm.nih.gov/geo/query/acc.cgi?acc=GSE159515). BedGraph files are also accessible through the genome browser at ToxoDB.org.

## Author Contributions

BM and SL conceptualized and designed the study. BM conducted experiments and analyzed the data. BW supplied the conventional RNA-seq datasets and provided key technical advice. BM wrote the first draft of the manuscript. HL and SL acquired funding. SL supervised the study. All authors contributed to the article and approved the submitted version.

## Funding

This work was supported by a Boehringer Ingelheim Fonds PhD fellowship to BM and funds from the National Institutes of Health to SL (1R01AI144369) and HL (U19AI110819).

## Conflict of Interest

The authors declare that the research was conducted in the absence of any commercial or financial relationships that could be construed as a potential conflict of interest.
