# Supplementary material for: High-Resolution Mapping of Transcription Initiation in the Asexual Stages of Toxoplasma gondii
Source: Front Cell Infect Microbiol. 2021 Jan 20;10:617998. doi: 10.3389/fcimb.2020.617998 (PMC7854901; doi:10.3389/fcimb.2020.617998)
Supplement: Supplementary file 7 [file DataSheet_7.pdf]

SUPPLEMENTARY MATERIAL

Figure S1. Mapping promoter activity at nucleotide resolution

Figure S2. Outliers in RNA-seq versus RAMPAGE are frequently explained by erroneous gene models

Figure S3. Defining *Toxoplasma* transcription start sites and 5′ leaders

Figure S4. Empirical TSS predictions for 91% of protein-coding genes

Figure S5. Stage-specific alternative TSS usage in *Toxoplasma*

Figure S6. Nucleotide composition around transcriptional and translational start sites

Figure S7. Canonical sequence determinants for nucleosome positioning in *Toxoplasma* are lacking

Figure S8. Highly-symmetric nucleosomal arrays and patterns of transcription initiation

Figure S9. *Toxoplasma* 5′ leaders are unusually long and lack suppression of uAUGs and uORFs

Table S1. Manual inspection of loci from outliers in RNA-seq versus RAMPAGE (cumulative 5′-tag count)

Table S2. Genes with zero counts by RNA-seq and/or RAMPAGE (cumulative 5′-tag count)

Table S3. Manual inspection of loci randomly-sampled from candidates for alternative TSS usage

Data S1. TSV file compiling various metrics from this analysis for each of the 8,322 protein-coding genes

Data S2. BED file with TSS positions curated from Bz and Tz predictions for the ME49 genome (v.45)

Data S3. TSV file with details on the 682 candidates with stage-dependent alternative TSS usage

Data S4. TSV file with details on the 1,490 putative bidirectionally-paired genes

Data S5. BED file with uORF predictions in ME49 Tz, mapped in the ME49 genome (v.45)

Data S6. BED file with uORF predictions in ME49 Bz, mapped in the ME49 genome (v.45)

FIGURE S1

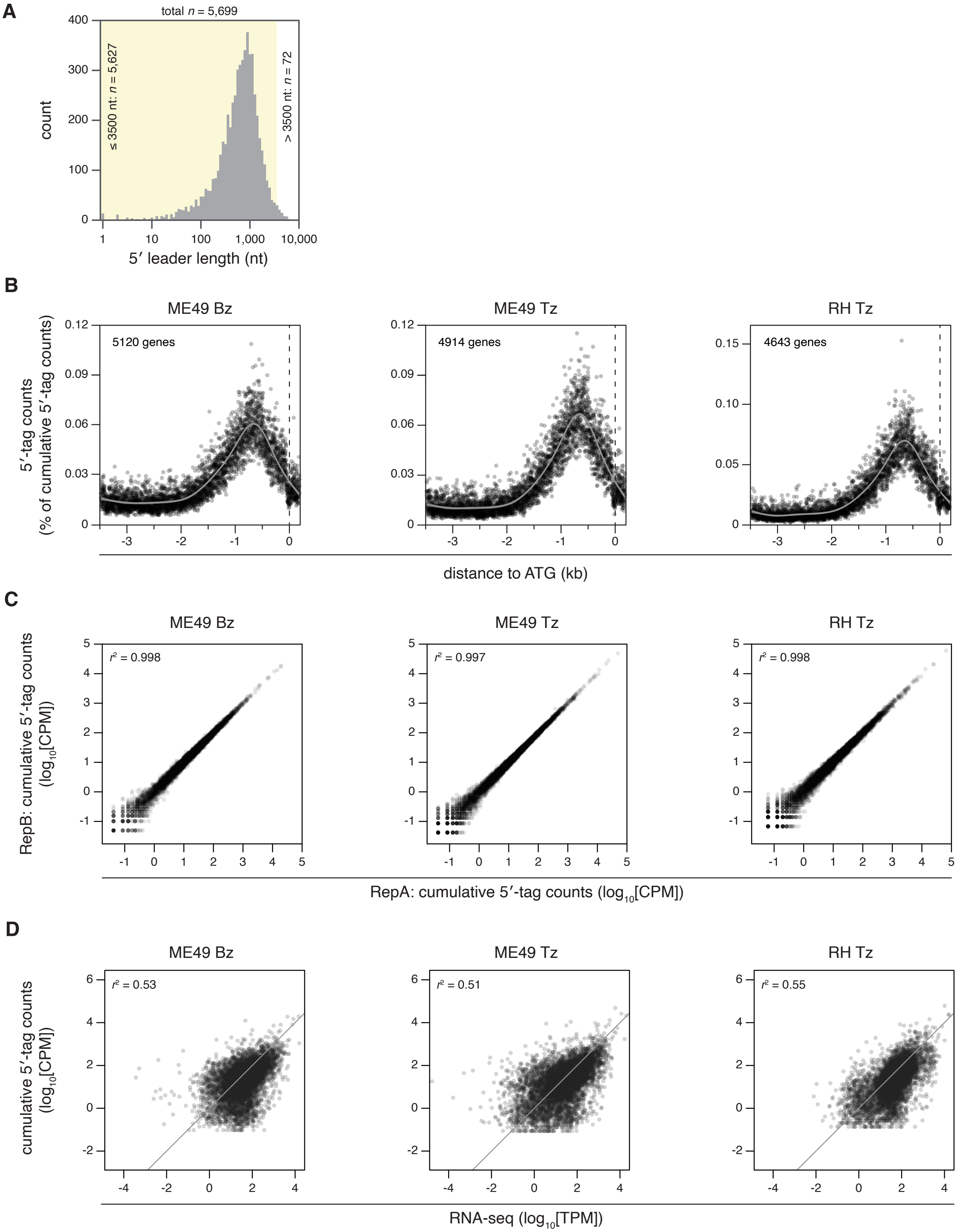
A
total n = 5,699
count
≤ 3500 nt: n = 5,627
> 3500 nt: n = 72
5′ leader length (nt)
B
ME49 Bz
ME49 Tz
RH Tz
5120 genes
4914 genes
4643 genes
5′-tag counts (% of cumulative 5′-tag counts)
distance to ATG (kb)
C
ME49 Bz
ME49 Tz
RH Tz
r² = 0.998
r² = 0.997
r² = 0.998
RepB: cumulative 5′-tag counts (log10[CPM])
RepA: cumulative 5′-tag counts (log10[CPM])
D
ME49 Bz
ME49 Tz
RH Tz
r² = 0.53
r² = 0.51
r² = 0.55
cumulative 5′-tag counts (log10[CPM])
RNA-seq (log10[TPM])

Figure S1. **Mapping promoter activity at nucleotide resolution**. (**A**) Length distribution of 5699 current models of *Toxoplasma* 5′ leaders (ME49 v.45). The number of 5′ leaders with lengths of 3500 nt or shorter, and with lengths greater than 3500 nt are indicated. (**B**) Relative 5′-tag density as a function of distance to annotated start codons. Relative 5′-tag densities were calculated for each nucleotide position within all gene-association windows individually, before averaging the relative 5′-tag densities across all windows. A trendline and 95% confidence interval were modelled. To prevent skewing of this data representation, this analysis was restricted to genes (i) with unshortened gene-association windows, and (ii) with cumulative 5′-tag counts above the 1st percentile of the respective dataset. (**C**) Reproducibility of cumulative 5′-tag counts between biological replicates. (**D**) Comparison of cumulative 5′-tag counts and standard RNA-seq for relative quantification of gene expression. Replicate-averaged 5′-tag counts and RNA-seq data were generated using samples of corresponding parasite strains and life-cycle stages (see materials and methods for details). For clarity, genes with zero counts in either dataset are not shown. CPM, counts per million. TPM, transcripts per million.

FIGURE S2

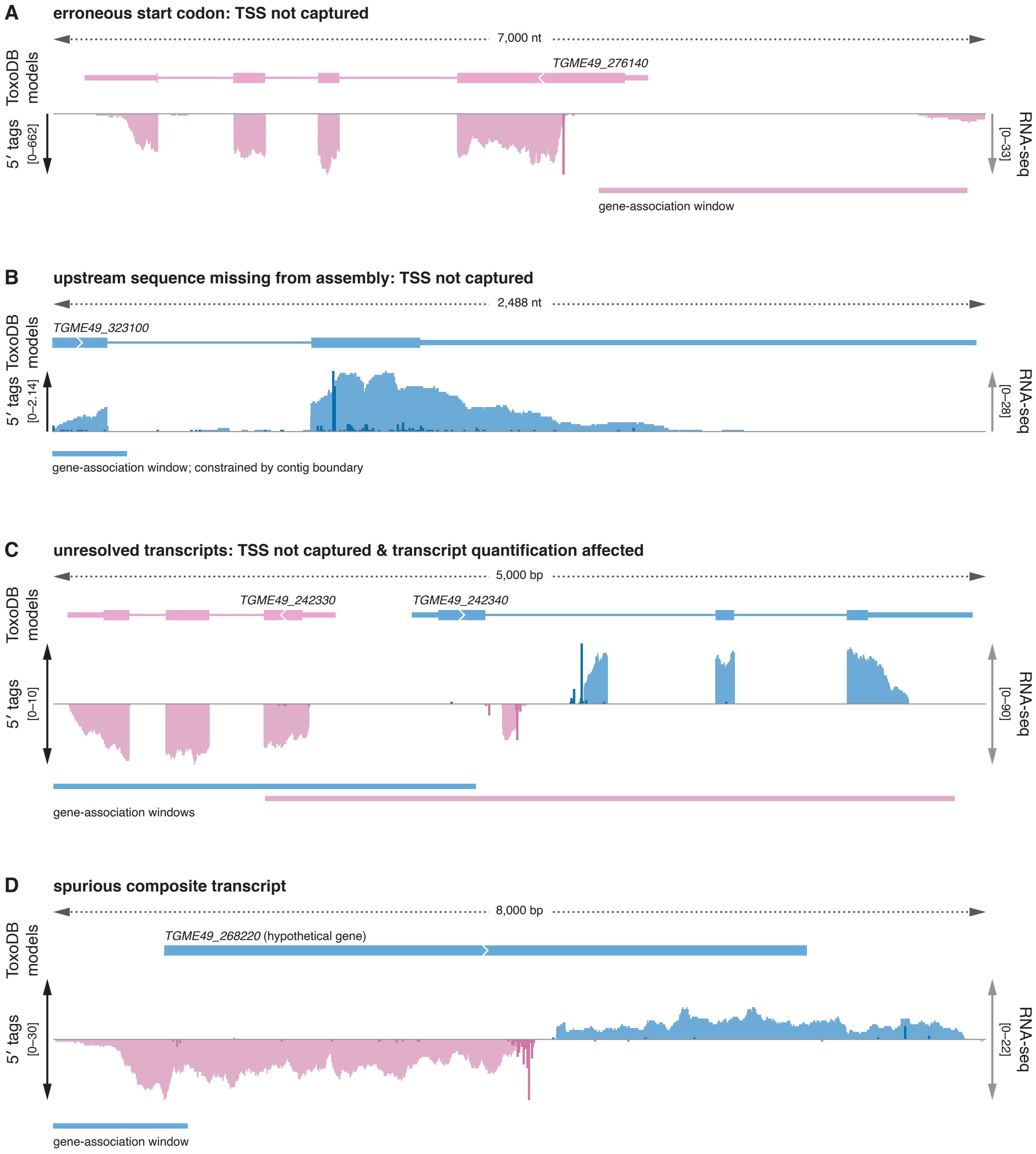

A
erroneous start codon: TSS not captured
7,000 nt
TGME49_276140
ToxoDB models
5′ tags [0–662]
RNA-seq [0–33]
gene-association window
B
upstream sequence missing from assembly: TSS not captured
2,488 nt
TGME49_323100
ToxoDB models
5′ tags [0–2.14]
RNA-seq [0–28]
gene-association window; constrained by contig boundary
C
unresolved transcripts: TSS not captured & transcript quantification affected
5,000 bp
TGME49_242330
TGME49_242340
ToxoDB models
5′ tags [0–10]
RNA-seq [0–90]
gene-association windows
D
spurious composite transcript
8,000 bp
TGME49_268220 (hypothetical gene)
ToxoDB models
5′ tags [0–30]
RNA-seq [0–22]
gene-association window

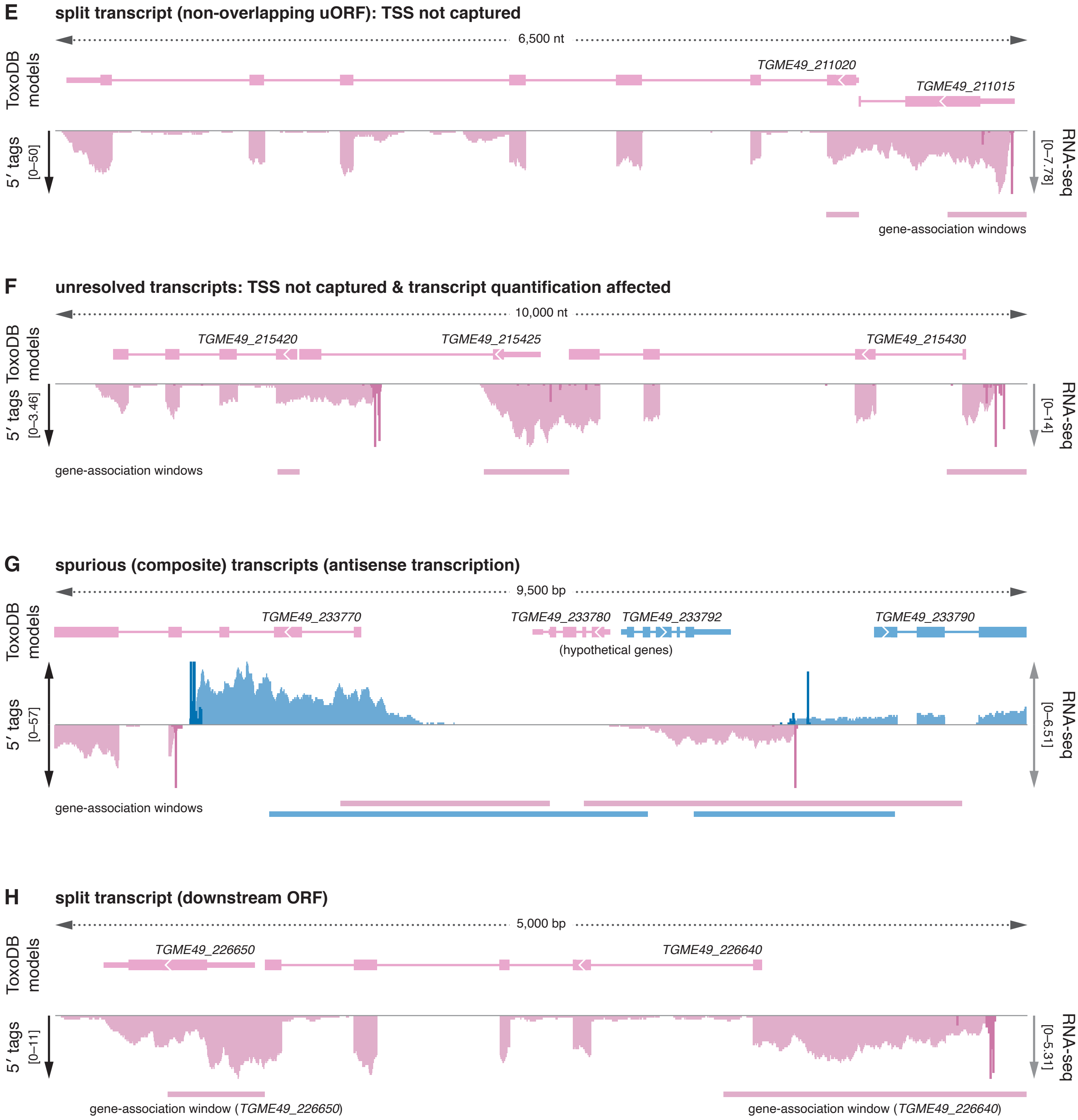

Figure S2. Outliers in RNA-seq versus RAMPAGE are frequently explained by erroneous gene models. (**A–H**) Examples of erroneous gene models or incompletely assembled loci, which prevent accurate transcript quantification by RNA-seq and/or cumulative 5′-tag counts.

FIGURE S3

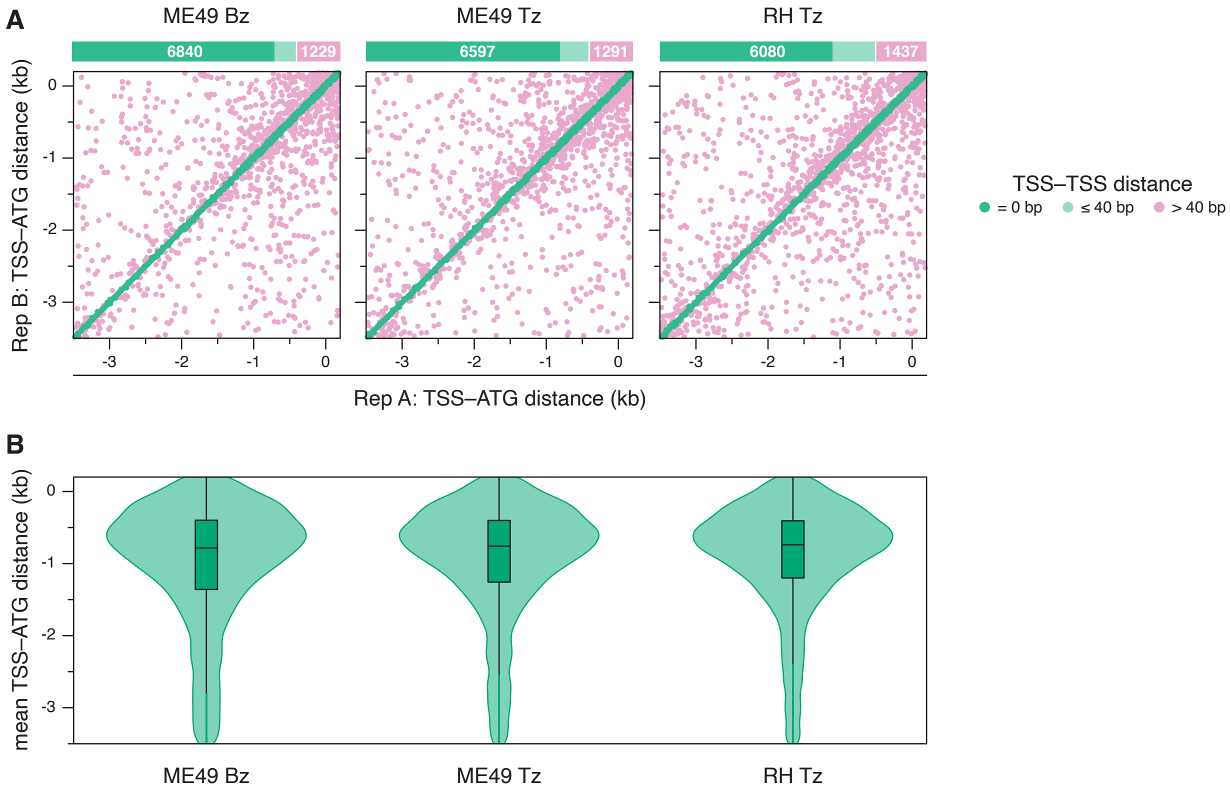

Figure S3. **Defining *Toxoplasma* transcription start sites and 5′ leaders**. (**A**) Genes for which TSSs were identified in both biological replicates, plotted by the distance to the annotated start codon. Genes are colored by the distance between replicate TSSs, and quantifications of these groups are represented as bars. (**B**) Violin and box plots showing the distribution of the mean distance between annotated start codons and TSSs replicable within 40 nt. The rounded geometric center between reproducible TSSs was used to calculate the mean distance to the start codon.

FIGURE S4

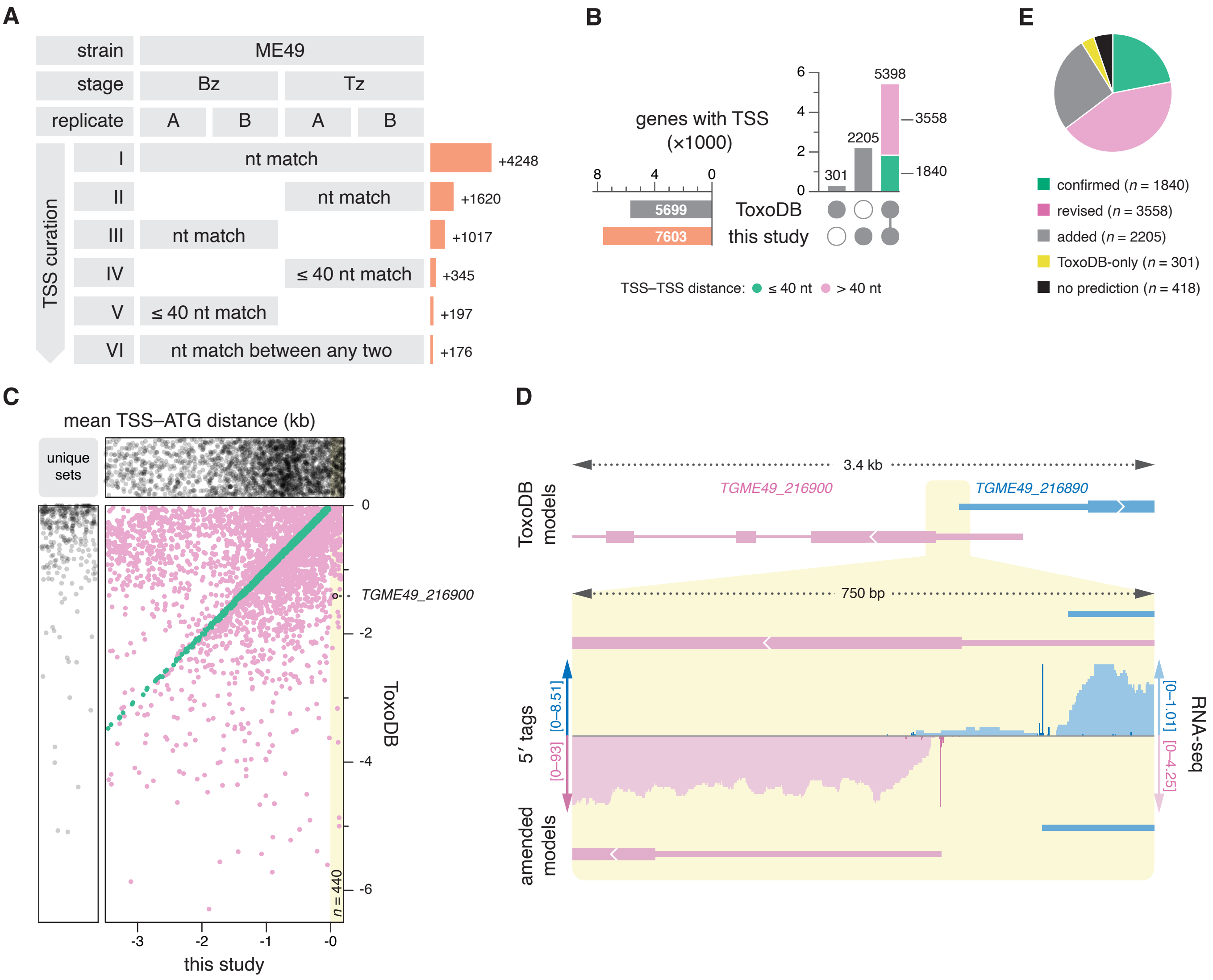

Figure S4. **Empirical TSS predictions for 91% of protein-coding genes**. (**A**) Successive approach for the curation of a Tz-biased prediction of *Toxoplasma* TSSs and 5′-leader lengths. (**B**) UpSet plot shows the intersection of TSSs from ToxoDB and this study, colored by inter-TSS distance. TSSs separated by 40 nt or less were considered congruent for the purpose of this comparison. (**C**) Genes with predicted TSSs in both datasets, or with a prediction only present in one dataset, plotted by distance between TSS and annotated start codon. Genes are colored according to the distance between their TSS predictions. The negative range on this study's scale is colored in yellow to demarcate genes whose TSSs are predicted to fall into the ORF. (**D**) Example of a gene (*TGME49_216900*) with a category I TSS prediction that falls into the annotated ORF. 5′-tag and RNA-seq count densities were used to manually amend ToxoDB gene models, resulting in a truncated first exon of *TGME49_216900* and new predictions for the 5′ leaders of *TGME49_216900* and *TGME49_216890*. Data ranges of respective datasets are indicated in CPM or TPM, respectively. (**E**) Pie chart representing the 8322 protein-coding genes of ME49, and the fractions of TSS predictions contributed by ToxoDB and the present study.

FIGURE S5

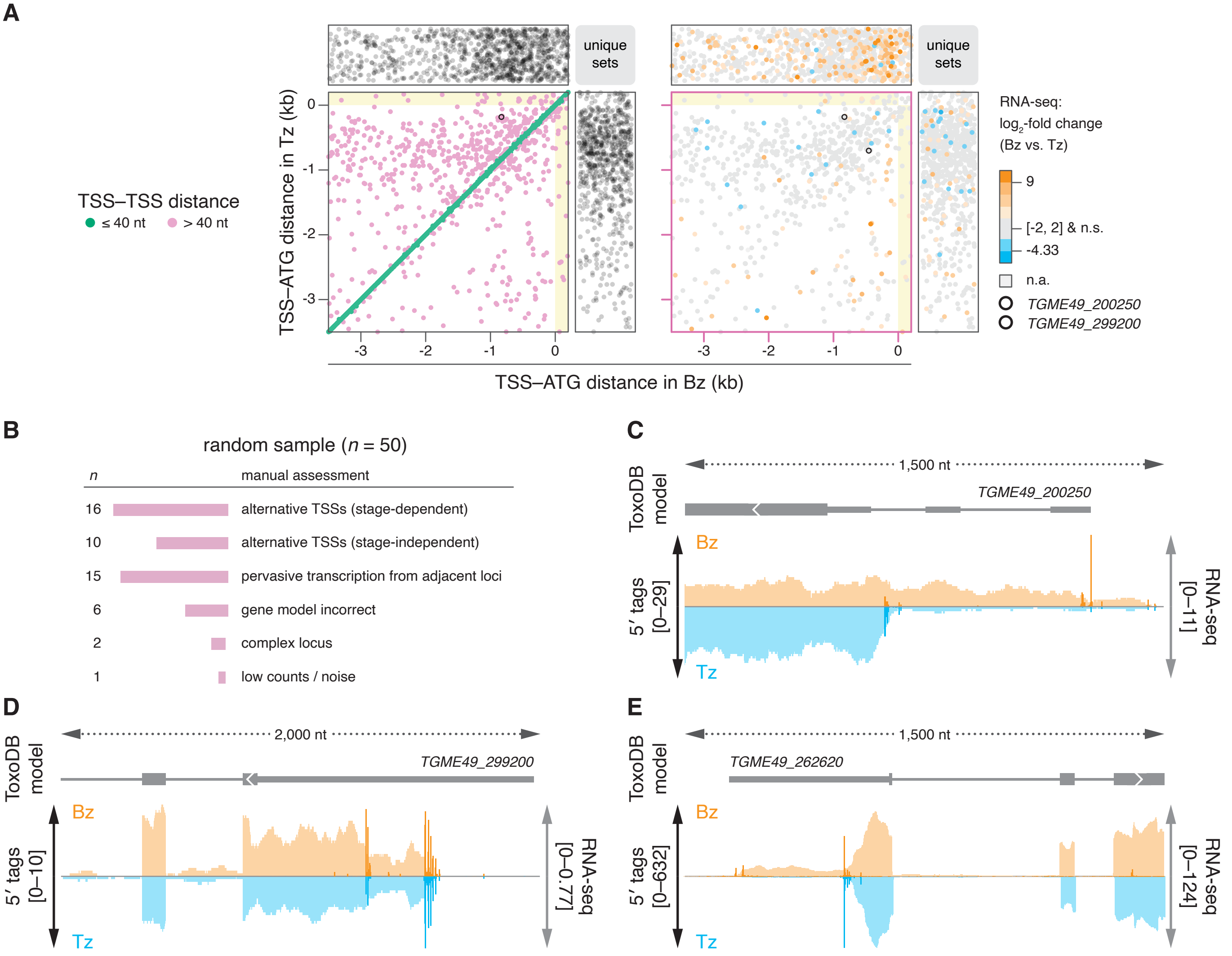

Figure S5. **Stage-specific alternative TSS usage in *Toxoplasma***. (**A**) Genes with nucleotide-matching TSSs between biological replicates in both ME49 Bz and Tz, plotted by TSS distance to the start codon for sets identified in both, or only one of the life-cycle stages (unique sets). Left, genes are colored according to the distance between TSSs from ME49 Bz and Tz. Right, genes are colored according to differential expression in Bz compared to Tz (log_2 scale). n.s., not significant. n.a., not applicable, i.e. genes below the coverage threshold required to calculate statistical significance. Genes with inter-TSS distances of 40 nt and below were omitted for clarity. The yellow-shaded ranges demarcate genes for which one the TSS shifts into the gene's ORF in at least one life-cycle stage. (**B**) Results from manual inspection of 50 loci randomly-sampled from genes with stage-dependent alternative TSS predictions. (**C**) Example of a gene (*TGME49_200250*) for which the dominant TSS shifts stage-specifically; here, resulting in a 649-nt extension of the 5′ leader in Bz. (**D**) Example of a gene (*TGME49_299200*) with alternative (stage-independent) TSS usage; here, generating transcript isoforms with distinct 5′ leaders. (**E**) Example of a gene (*TGME49_262620*) for which a minor TSS appears to be regulated stage-dependently; here, resulting in a transcript isoform with a 317-nt extension of the 5′ leader in Bz. Data ranges of respective datasets are indicated in CPM and TPM respectively. Read coverage is only shown for the relevant strand.

FIGURE S6

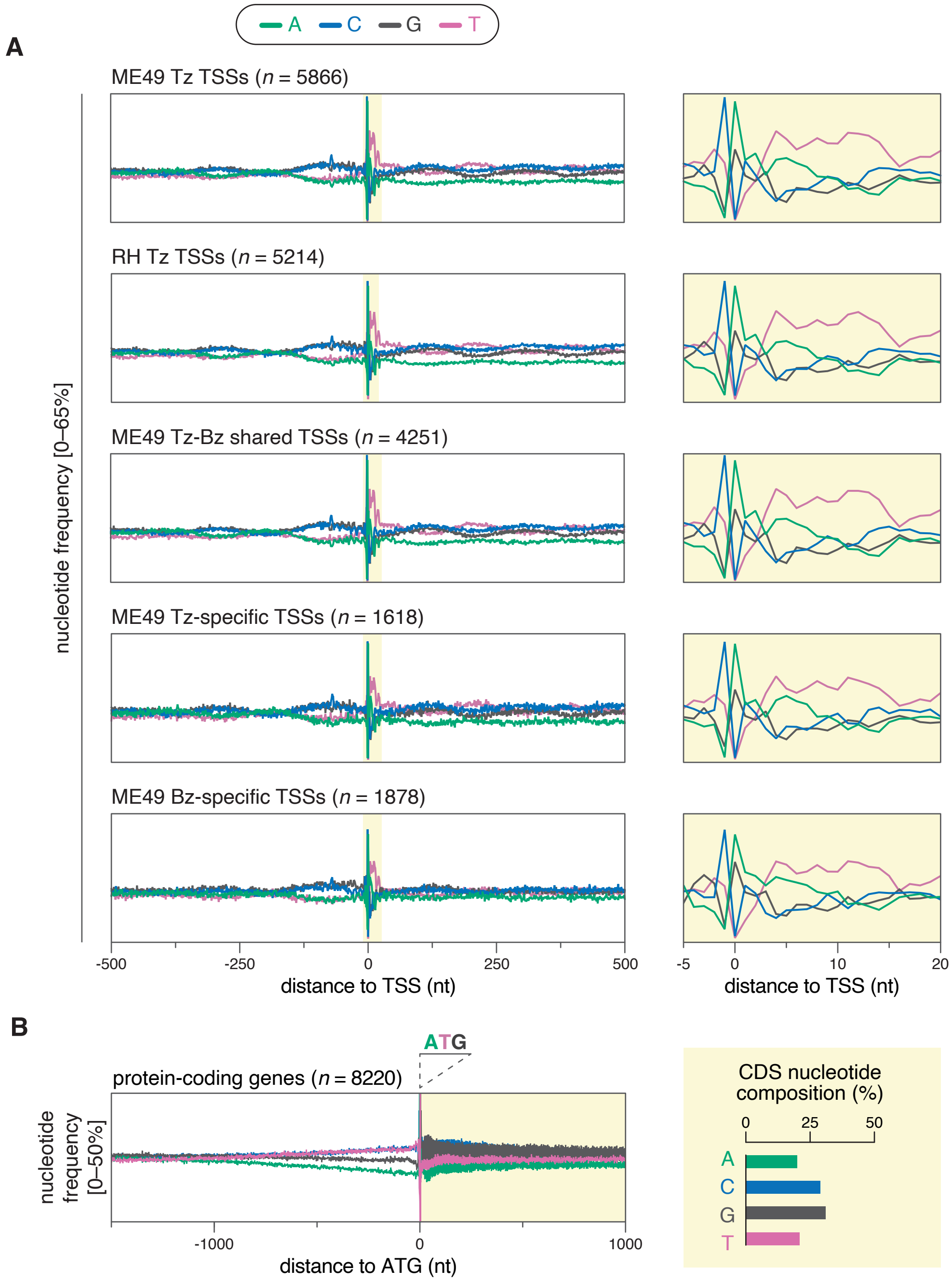

Figure S6. **Nucleotide composition around transcriptional and translational start sites**. (**A**) Nucleotide composition around different subsets of TSSs that were reproducible at the nucleotide position. For subsetting, a 40-nt cutoff was used to evaluate TSSs for sharedness (if within 40 nt) versus specificity (if farther than 40 nt) between samples. (**B**) Nucleotide composition around the start codons of all protein-coding genes (ME49 v.45). The average nucleotide composition within the CDS is shown on the right. Nucleotide frequencies within CDSs were calculated from codon- and amino-acid-frequency tables available on ToxoDB.org.

FIGURE S7

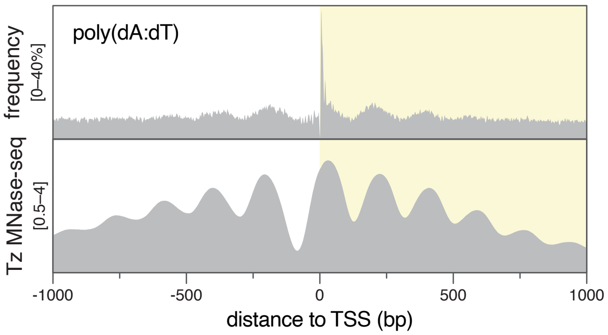

Figure S7. **Canonical sequence determinants for nucleosome positioning in *Toxoplasma* are lacking**. Frequency of poly(dA:dT) tracts and nucleosome occupancy around nt-matching TSSs in ME49 Tz. Stretches of at least four consecutive deoxyadenosines or deoxythymidines were evaluated. The concentration of poly(dA:dT) just downstream of TSSs corresponds to the downstream thymidine cluster.

FIGURE S8

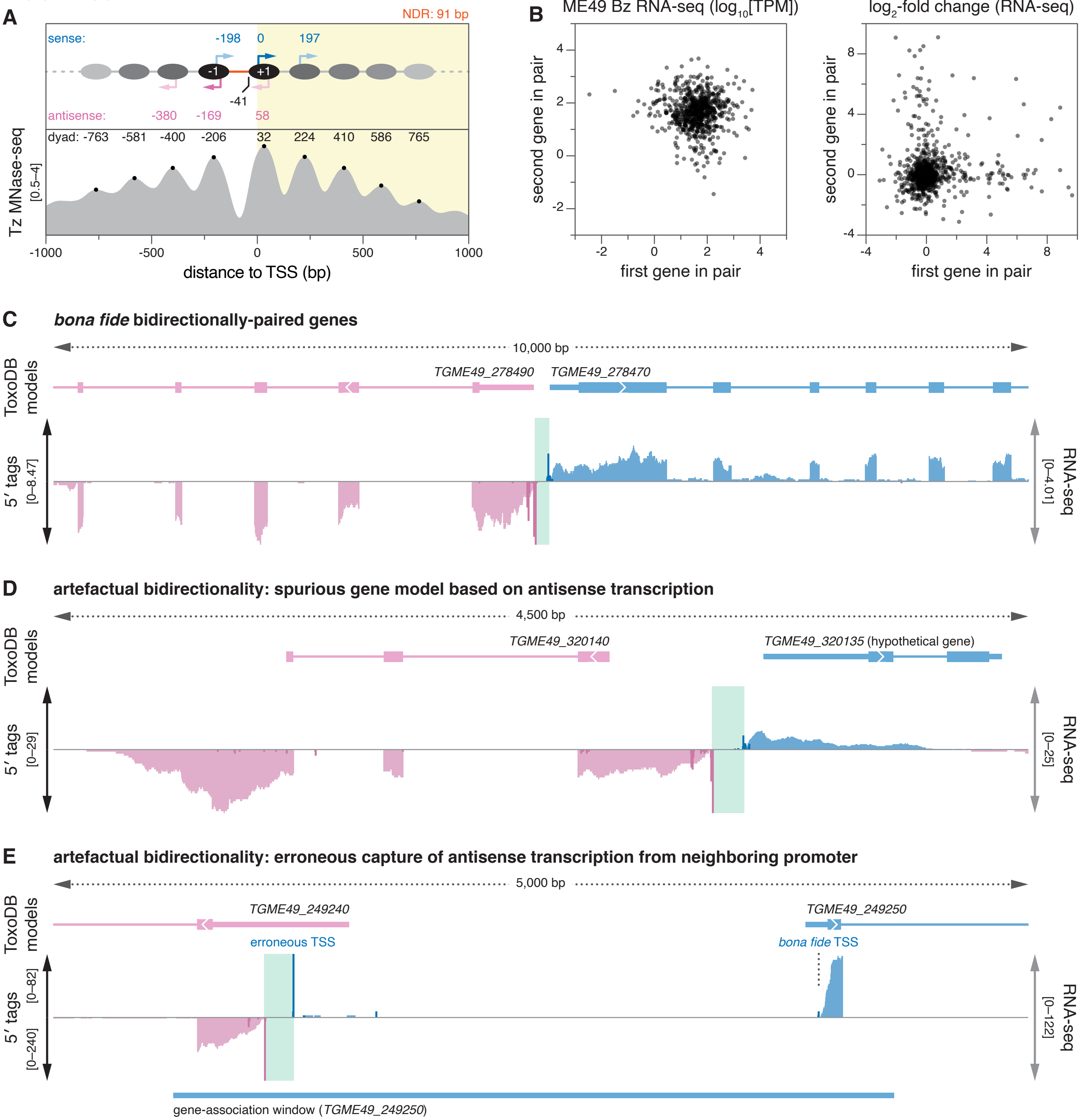

Figure S8. **Highly-symmetric nucleosomal arrays and patterns of transcription initiation**. (**A**) Nucleosome occupancy around nucleotide-matching TSSs in ME49 Tz. Top, schematic representation of nucleosome phasing. Bottom, nucleosome occupancy as determined by MNase-seq on Tz of the *Toxoplasma* Pru strain (Farhat et al., 2020). Peak densities are indicated, which are expected to correspond to the nucleosome dyad. A length of 147 bp of nucleosomal DNA was assumed to calculate the geometries of the NDR and the upstream edge of the +1 nucleosome. Sites of peak 5′-tag densities in sense and antisense orientation are indicated. (**B**) Evaluation of co-expression of bidirectionally-paired genes. Left, RNA-seq expression data. Right, differential expression in ME49 Bz compared to Tz. (**C–E**) Categorical examples of gene pairs predicted to be bidirectionally paired purely based on inter-TSS distance (green-shaded range). Most predictions represent *bona fide* bidirectionally-paired genes; however, artefacts were also captured.

FIGURE S9

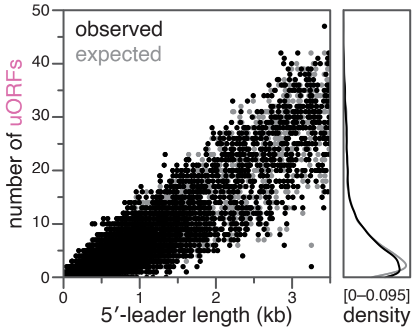

Figure S9. ***Toxoplasma* 5′ leaders are unusually long and lack suppression of uAUGs and uORFs**. Left, scatter plot showing the number of uORFs per gene as a function of 5′-leader length in ME49 Tz. Right, corresponding density plot.

Table S1. Manual inspection of loci from outliers in RNA-seq versus RAMPAGE (cumulative 5′-tag count)

	Gene ID	RNA-seq, log_{10}(TPM)	cumulative 5′-tag count, log_{10}(CPM)	window size (nt)	assessment
overquantified by cumulative 5′-tag count	TGME49_201775	0.545407079302844	3.96019534495458	3700	gene model incorrect
	TGME49_202960	-4.88605664769316	1.21772768182157	3700	discrepancy unexplained
	TGME49_205760	-1.30120374820957	1.41132390150899	3700	discrepancy unexplained
	TGME49_207740	-1.37383420686072	1.26221013749928	3700	discrepancy unexplained
	TGME49_209300	-0.144660777641917	2.43942120089404	3700	gene model incorrect
	TGME49_210210	-2.5777385491864	-0.0325819590778681	3700	gene model incorrect
	TGME49_210970	-2.15651805696004	2.09502331278254	449	gene model incorrect
	TGME49_215970	-1.60696910248558	1.08719907828394	922	gene model incorrect
	TGME49_215980	0.719123787213718	3.33616001408002	1805	discrepancy unexplained
	TGME49_218510	-2.11798803837334	2.3481023802157	3700	gene model incorrect
	TGME49_224675	-0.680802300684495	1.84834090894041	3700	gene model incorrect
	TGME49_226230	-1.14825869084897	2.09536170103704	3700	gene model incorrect
	TGME49_227070	-1.5443634650524	1.08422941734902	3700	gene model incorrect
	TGME49_229230	-0.677966871368197	2.08667341008926	3700	discrepancy unexplained
	TGME49_229270	-1.56567066266227	1.14457899298432	3700	discrepancy unexplained
	TGME49_231060	-1.2804035759617	1.7471615122046	3700	discrepancy unexplained
	TGME49_234165	-1.00783102285875	1.91851353044596	3700	gene model incorrect
	TGME49_237080	-0.676438536537133	2.01539780635732	3700	gene model incorrect
	TGME49_238980	-1.56120458427868	1.89144870640784	3700	gene model incorrect
	TGME49_239430	-0.441931345318517	2.89251825922695	3700	gene model incorrect
	TGME49_240550	-1.28878347567891	2.27570733370858	3700	discrepancy unexplained
	TGME49_242360	-1.94149805657035	1.16030941757332	3700	gene model incorrect
	TGME49_246555	-1.26544017842052	1.36640213158771	3700	gene model incorrect
	TGME49_247980	-2.43758816705027	0.683843324073751	3700	discrepancy unexplained
	TGME49_249960	-0.885619232837273	1.73064511838049	3700	gene model incorrect
	TGME49_251870	0.0736358815389812	3.0503976273235	1752	histone H2Bb
	TGME49_259680	-2.94385773794095	1.73400739188294	3700	gene model incorrect
	TGME49_262020	-3.87942606879415	0.441086484185919	3700	gene model incorrect
	TGME49_266650	-1.83511204245205	1.56863192448032	3700	gene model incorrect
	TGME49_266980	-1.14462018011366	2.56930424493347	3700	gene model incorrect
	TGME49_269365	-3.05998184499234	-0.0977488433245003	3700	gene model incorrect
	TGME49_272265	-1.26901596150448	1.66904239885736	3700	gene model incorrect
	TGME49_273080	0.28629700454752	2.89132966695524	3700	gene model incorrect
	TGME49_275680	-3.36957212497498	1.60582759151077	3700	discrepancy unexplained
	TGME49_281040	-1.62280306499109	1.16886171238435	3700	gene model incorrect
	TGME49_289930	-0.625255318937697	2.04981130577613	3700	gene model incorrect
	TGME49_294300	-1.36917564360299	1.73000045473352	3553	gene model incorrect
	TGME49_295640	-0.775135580867005	1.8553629916587	3700	gene model incorrect
	TGME49_305890	-0.383908010874351	2.51308978482482	3700	gene model incorrect
	TGME49_306500	-1.07342605996739	1.52987534515643	3700	gene model incorrect
	TGME49_316320	-1.07981403959885	1.67241693704253	3700	discrepancy unexplained
	TGME49_316390	-1.48190739848347	1.27325347495561	3700	gene model incorrect
	TGME49_320290	-1.5587291565795	2.18836952058182	3700	gene model incorrect
underquantified by cumulative 5′-tag count	TGME49_206690	2.24801283933676	-0.36530544417335	226	gene model incorrect
	TGME49_207060	2.84174266323001	0.289205298577194	486	gene model incorrect
	TGME49_211000	1.72032566836586	-0.986824468516077	714	gene model incorrect
	TGME49_211020	2.57254928488953	-0.310733209460723	218	gene model incorrect
	TGME49_213635	2.23872839580758	-0.637339466348067	3700	gene model incorrect
	TGME49_213890	1.51878640707841	-1.13733946634836	240	gene model incorrect
	TGME49_215040	1.61974435722528	-1.13733946634836	266	gene model incorrect
	TGME49_215420	2.26861597332732	-0.549293836820026	225	gene model incorrect
	TGME49_216415	1.51851092612694	-1.13733946634836	770	gene model incorrect
	TGME49_217610	2.05150984030733	-0.748263841156546	2199	gene model incorrect
	TGME49_217951	2.70611962952636	0.165313056721637	3700	gene model incorrect
	TGME49_218910	1.63850879477495	-1.13733946634836	3700	gene model incorrect
	TGME49_219690	2.40501377642226	-0.549293836820226	1955	gene model incorrect
	TGME49_220580	1.76716501215342	-1.13733946634836	3700	gene model incorrect
	TGME49_221522	2.14449443953444	-0.509703213796715	220	gene model incorrect
	TGME49_221910	2.67574515077857	0.00878856933136626	559	gene model incorrect
	TGME49_225510	2.12350356959894	-0.535279475020774	910	gene model incorrect
	TGME49_226650	2.4954894561842	-0.685794472852396	499	gene model incorrect
	TGME49_231180	1.75008019934569	-0.836309470684387	201	gene model incorrect
	TGME49_232035	2.01647397123378	-0.748263841156044	249	gene model incorrect
	TGME49_233770	2.00092639933342	-0.89877883898833	2047	gene model incorrect
	TGME49_240240	1.62294016954721	-0.986824468516077	2561	gene model incorrect
	TGME49_240250	2.24349339225299	-0.748263841156044	215	gene model incorrect
	TGME49_240800	2.19161241681444	-0.509703213796715	3700	gene model incorrect
	TGME49_242340	3.15984139399613	-0.0642754485084687	3700	gene model incorrect
	TGME49_244320	2.63989412034666	-0.0435791059295785	1863	gene model incorrect
	TGME49_246000	2.29949347209561	-0.359188215964474	612	gene model incorrect
	TGME49_246178	2.05793812967121	-0.549293836820226	249	gene model incorrect
	TGME49_248440	1.72602052696293	-0.836309470684682	989	gene model incorrect
	TGME49_248880	2.72033244980805	-0.36530544417335	3700	gene model incorrect
	TGME49_253930	1.71349737246798	-0.986824468516077	1599	gene model incorrect
	TGME49_254606	2.49623965914366	-0.447233845492565	554	gene model incorrect
	TGME49_258040	1.72382548581932	-0.89877883898833	1014	gene model incorrect
	TGME49_258140	1.97335952024666	-0.748263841156841	3700	gene model incorrect
	TGME49_261070	2.39480692497413	-0.165098130273552	825	gene model incorrect
	TGME49_262130	1.83447005656929	-0.685794472852396	328	gene model incorrect
	TGME49_265470	2.11837838192575	-0.535279475020406	426	gene model incorrect
	TGME49_267610	2.39867702690674	-0.160218211628833	1960	gene model incorrect
	TGME49_268220	2.47827552328024	-0.398778838988236	3700	gene model incorrect
	TGME49_270830	2.8041351459572	-0.227567498577702	3700	gene model incorrect
	TGME49_271450	2.01109086918896	-0.685794472852396	1925	gene model incorrect
	TGME49_272600	2.32289605767543	-0.310733209460723	1011	gene model incorrect
	TGME49_276140	2.96264215597143	0.141414134603599	2768	gene model incorrect
	TGME49_283900	1.74199202381078	-0.836309470684682	1502	gene model incorrect
	TGME49_284598	2.2572619490718	-0.263245452845689	272	gene model incorrect
	TGME49_287450	2.0177697540869	-0.597748843324556	710	gene model incorrect
	TGME49_290740	1.64000604678911	-0.986824468516077	763	gene model incorrect
	TGME49_291840	2.92923611211064	-0.336309470684454	3700	gene model incorrect
	TGME49_293400	1.99029723068481	-0.748263841156044	438	gene model incorrect
	TGME49_294750	1.92727779608832	-0.597748843324556	375	gene model incorrect
	TGME49_294990	2.73758376347431	-0.248263841156295	410	gene model incorrect
	TGME49_301370	1.69005828046066	-0.898778838988236	1317	gene model incorrect
	TGME49_304680	1.9486425089693	-0.986824468516672	592	gene model incorrect

Gene ID	RNA-seq, log_{10}(TPM)	cumulative 5′-tag count, log_{10}(CPM)	window size (nt)	assessment
TGME49_312220	2.36435884648799	-0.271142586437208	701	gene model incorrect
TGME49_314660	1.98884211016538	-1.13733946634836	1308	gene model incorrect
TGME49_315798	1.78604201343783	-0.986824468516077	466	gene model incorrect
TGME49_320600	3.23183708276885	0.497650371974509	3700	gene model incorrect
TGME49_323100	3.17806009395287	-0.0246848254846072	200	gene model incorrect
TGME49_324600	2.75705088039801	-0.836309470684387	262	gene model incorrect
TGME49_325800	3.06809056114225	-0.748263841156737	3700	gene model incorrect

Table S2. Genes with zero counts by RNA-seq and/or RAMPAGE (cumulative 5′-tag count)

Gene ID	RNA-seq (TPM)	cumulative 5′-tag count (CPM)	window size (nt)	gene description
TGME49_210100	0	0.03606513	946	hypothetical protein
TGME49_238470	0	0.03606513	2646	SAG-related sequence SRS22C
TGME49_238500	0	0.03606513	3211	SAG-related sequence SRS22F
TGME49_265065	0	0.03606513	518	hypothetical protein
TGME49_281555	0	0.03606513	3700	hypothetical protein
TGME49_285160	0	0.03606513	3700	cyclin-dependent kinase family 5 protein
TGME49_315380	0	0.03606513	2366	SAG-related sequence SRS53B
TGME49_216150	0	0.036827613	637	peptidase family M3 protein
TGME49_238860	0	0.036827613	3700	hypothetical protein
TGME49_259300	0	0.036827613	3700	SAG-related sequence SRS26B
TGME49_263639	0	0.036827613	370	hypothetical protein
TGME49_297300	0	0.036827613	3700	hypothetical protein
TGME49_329620	0	0.07213026	2403	hypothetical protein
TGME49_329710	0	0.07213026	1970	SAG-related sequence SRS26I
TGME49_295300	0	0.072892743	3700	Toxoplasma gondii family B protein
TGME49_297380	0	0.072892743	2085	hypothetical protein
TGME49_202330	0	0.108957873	3700	hypothetical protein
TGME49_206910	0	0.108957873	625	hypothetical protein
TGME49_209583	0	0.108957873	844	hypothetical protein
TGME49_283480	0	0.108957873	3700	cell-cycle-associated protein kinase DYRK2, putative
TGME49_301190	0	0.108957873	342	hypothetical protein
TGME49_214160	0	0.109720357	3700	hypothetical protein
TGME49_217390	0	0.109720357	3700	hypothetical protein
TGME49_202990	0	0.11048284	3700	hypothetical protein
TGME49_259900	0	0.11048284	3311	hypothetical protein
TGME49_284610	0	0.11048284	3700	hypothetical protein
TGME49_282020	0	0.145023003	3700	hypothetical protein
TGME49_200110	0	0.145785487	3700	hypothetical protein
TGME49_200600	0	0.145785487	3700	hypothetical protein
TGME49_238505	0	0.145785487	3700	hypothetical protein
TGME49_259895	0	0.14654797	3700	hypothetical protein
TGME49_272560	0	0.147310454	3700	SAG-related sequence SRS31B
TGME49_244890	0	0.180325649	3700	hypothetical protein
TGME49_211060	0	0.181088133	290	hypothetical protein
TGME49_270080	0	0.181850616	509	hypothetical protein
TGME49_287990	0	0.181850616	3700	hypothetical protein
TGME49_242600	0	0.1826131	2844	6-phosphogluconate dehydrogenase
TGME49_274700	0	0.21867823	3700	hypothetical protein
TGME49_311820	0	0.21867823	3700	hypothetical protein
TGME49_297440	0	0.255505843	3700	hypothetical protein
TGME49_202735	0	0.256268327	3700	hypothetical protein
TGME49_230667	0	0.256268327	3700	hypothetical protein
TGME49_243550	0	0.256268327	3700	H-SHIPPO 1, putative
TGME49_268940	0	0.290046006	3700	hypothetical protein
TGME49_227040	0	0.29080849	3700	hypothetical protein
TGME49_215185	0	0.292333457	721	hypothetical protein
TGME49_257925	0	0.328398587	3700	hypothetical protein
TGME49_272360	0	0.362938749	2544	EF hand family protein
TGME49_315345	0	0.362938749	2742	SAG-related sequence SRS52F
TGME49_210815	0	0.3652262	3700	hypothetical protein
TGME49_259410	0	0.365988683	3700	SAG-related sequence SRS26A
TGME49_315390	0	0.366751167	2464	SAG-related sequence SRS53C
TGME49_286170	0	0.399003879	3700	hypothetical protein
TGME49_243220	0	0.399766363	3700	hypothetical protein
TGME49_208770	0	0.400528846	3700	hypothetical protein
TGME49_217800	0	0.436593976	3700	hypothetical protein
TGME49_307600	0	0.438118943	3700	RCC1 domain containing protein
TGME49_304980	0	0.511011686	3700	hypothetical protein
TGME49_243378	0	0.580854496	3700	hypothetical protein
TGME49_268270	0	0.617682109	3700	hypothetical protein
TGME49_307640	0	0.693624786	3023	CMGC kinase, CK2 family
TGME49_307450	0	0.764230079	3700	hypothetical protein
TGME49_214860	0	0.765755046	3700	hypothetical protein
TGME49_242300	0	0.798007758	2703	dihydrodipicolinate synthase
TGME49_255490	0	0.801057693	3700	hypothetical protein
TGME49_245740	0	0.80487011	2970	hypothetical protein
TGME49_247940	0	0.875475403	3700	hypothetical protein
TGME49_201895	0	0.910778049	3700	hypothetical protein
TGME49_236975	0	0.912303016	3700	Toxoplasma gondii family D protein
TGME49_236830	0	0.945318212	3700	hypothetical protein
TGME49_286465	0	0.982908309	548	hypothetical protein
TGME49_312850	0	0.984433276	3700	hypothetical protein
TGME49_240480	0	1.020498406	705	cpw-wpc domain-containing protein
TGME49_283495	0	1.061138437	3700	hypothetical protein
TGME49_278400	0	1.096441083	1904	Toxoplasma gondii family A protein
TGME49_300090	0	1.166283892	3700	hypothetical protein
TGME49_202210	0	1.344322091	975	nucleoside diphosphate kinase
TGME49_207407	0	1.348134509	981	hypothetical protein
TGME49_262575	0	1.351946927	3700	hypothetical protein
TGME49_298080	0	1.454042448	3700	hypothetical protein
TGME49_255200	0	1.457092382	3700	Radial spoke head protein 9, putative
TGME49_208380	0	1.458617349	3700	hypothetical protein
TGME49_219780	0	1.744088454	3700	hypothetical protein
TGME49_211080	0	1.781678551	3700	hypothetical protein
TGME49_200595	0	1.79006587	3700	hypothetical protein
TGME49_315855	0	1.928989004	3700	hypothetical protein
TGME49_278290	0	1.929751488	3700	Toxoplasma gondii family A protein
TGME49_244900	0	2.001881748	3576	dynein light chain type 1, putative
TGME49_205425	0	2.150717168	3700	hypothetical protein
TGME49_321280	0	2.588073628	3700	hypothetical protein
TGME49_307080	0	2.621851307	3700	hypothetical protein
TGME49_242370	0	2.700081435	3700	hypothetical protein
TGME49_305830	0	2.700843919	1868	EF hand domain-containing protein
TGME49_220850	0	3.200418352	3700	hypothetical protein
TGME49_286778	0	3.976848168	3700	hypothetical protein
TGME49_294030	0	4.326062214	3700	hypothetical protein

Gene ID	RNA-seq (TPM)	cumulative 5′-tag count (CPM)	window size (nt)	gene description
TGME49_264875	0	4.734978379	3700	hypothetical protein
TGME49_235590	0	5.029599286	3700	hypothetical protein
TGME49_275700	0	5.034936671	3700	hypothetical protein
TGME49_225435	0	5.239890197	3700	hypothetical protein
TGME49_313025	0	5.499208458	3239	hypothetical protein
TGME49_248970	0	5.982542333	3700	leucine rich repeat-containing protein
TGME49_307060	0	6.70155748	3700	hypothetical protein
TGME49_214115	0	6.752872281	3700	hypothetical protein
TGME49_328900	0	8.167037181	3700	hypothetical protein
TGME49_266710	0	9.24746611	3700	NEK kinase
TGME49_211070	0	9.432366661	430	hypothetical protein
TGME49_307840	0	9.47910656	3176	SF-assemblin/beta giardin protein
TGME49_242400	0	10.27482687	3700	calcium-dependent protein kinase CDPK2B
TGME49_312460	0	11.01442907	3700	zinc finger, MYND-type containing 12 family protein
TGME49_259965	0	11.0504942	3003	Bardet-Biedl syndrome 5, putative
TGME49_224040	0	11.48960403	3700	flagellar associated protein
TGME49_217710	0	12.20686581	3700	DnaJ domain-containing protein
TGME49_215650	0	12.49004946	3700	hypothetical protein
TGME49_242710	0	13.40845235	3700	hypothetical protein
TGME49_235670	0	15.26683606	3700	hypothetical protein
TGME49_231605	0	16.39019247	3700	hypothetical protein
TGME49_294260	0	16.61039566	2946	NEK kinase
TGME49_310455	0	17.89859965	3700	hypothetical protein
TGME49_204472	0	17.99535779	3700	cyclic nucleotide-binding domain-containing protein
TGME49_272905	0	18.63667689	3629	hypothetical protein
TGME49_266660	0	19.75416183	3700	mediator complex subunit MED9
TGME49_224905	0	20.00181444	3700	hypothetical protein
TGME49_244360	0	22.04891112	3700	hypothetical protein
TGME49_310215	0	22.09587942	3700	hypothetical protein
TGME49_251420	0	23.53444379	3700	hypothetical protein
TGME49_246763	0	24.09219538	3700	hypothetical protein
TGME49_301260	0	24.10210766	3700	hypothetical protein
TGME49_286570	0	24.54197998	3700	hypothetical protein
TGME49_320105	0	24.95242111	3700	hypothetical protein
TGME49_221240	0	29.15557234	3700	hypothetical protein
TGME49_202910	0	30.68555747	3700	zinc carboxypeptidase superfamily protein
TGME49_258575	0	31.03934642	3700	Dpy-30 motif protein
TGME49_254090	0	40.02901923	3700	hypothetical protein
TGME49_309185	0	42.44111371	3700	hypothetical protein
TGME49_226245	0	61.21236723	3700	hypothetical protein
TGME49_241000	0	86.13962639	3700	hypothetical protein
TGME49_304755	0	90.54750274	3700	hypothetical protein
TGME49_297390	0	126.6898753	3700	hypothetical protein
TGME49_320135	0	137.4474247	3700	hypothetical protein
TGME49_308970	0	171.575793	3612	hypothetical protein
TGME49_321460	0	210.6028418	3700	hypothetical protein
TGME49_214930	0	225.9261016	3700	hypothetical protein
TGME49_261390	0	371.3697706	3700	hypothetical protein
TGME49_281350	0.005554	0	3700	hypothetical protein
TGME49_224790	0.00634	0	1733	SAG-related sequence SRS40A
TGME49_322800	0.008477	0	3700	hypothetical protein
TGME49_234060	0.010388	0	366	hypothetical protein
TGME49_201100	0.012739	0	2228	hypothetical protein
TGME49_307590	0.013441	0	758	hypothetical protein
TGME49_317820	0.022587	0	3700	hypothetical protein
TGME49_224780	0.026744	0	2629	SAG-related sequence SRS40B
TGME49_243160	0.028034	0	2933	Toxoplasma gondii family A protein
TGME49_278350	0.034229	0	1744	Toxoplasma gondii family A protein
TGME49_326300	0.037883	0	201	hypothetical protein
TGME49_238450	0.042172	0	893	hypothetical protein
TGME49_325200	0.051737	0	3700	pyridoxal-phosphate dependent superfamily protein
TGME49_217380	0.05513	0	2550	hypothetical protein
TGME49_207730	0.059211	0	1927	hypothetical protein
TGME49_301998	0.068004	0	344	ORF C, putative
TGME49_313740	0.072341	0	3700	zinc finger (CCCH type) motif-containing protein
TGME49_307630	0.075356	0	3700	hypothetical protein
TGME49_201650	0.07623	0	293	hypothetical protein
TGME49_243180	0.092339	0	1971	Toxoplasma gondii family A protein
TGME49_326200	0.098581	0	522	type I fatty acid synthase
TGME49_240330	0.099392	0	3700	Toxoplasma gondii family E protein
TGME49_293368	0.101946	0	1000	TIP49 C-terminus family protein
TGME49_205658	0.104324	0	261	F5/8 type C domain-containing protein
TGME49_324200	0.10489	0	3700	hypothetical protein
TGME49_240350	0.105435	0	3700	Toxoplasma gondii family E protein
TGME49_278360	0.118366	0	1652	Toxoplasma gondii family A protein
TGME49_243100	0.124027	0	1738	Toxoplasma gondii family A protein
TGME49_238460	0.127745	0	3462	SAG-related sequence SRS22B
TGME49_321800	0.137202	0	3700	EGF family domain-containing protein
TGME49_204468	0.144099	0	685	hypothetical protein
TGME49_295750	0.14554	0	3700	IgA-specific serine endopeptidase
TGME49_203500	0.155961	0	2340	alanine dehydrogenase/pyridine nucleotide transhydrogenase domain-containing protein
TGME49_309550	0.178705	0	3700	hypothetical protein
TGME49_289040	0.186119	0	1201	Armadillo/beta-catenin family repeat-containing protein
TGME49_203570	0.19841	0	1107	acyltransferase domain-containing protein
TGME49_292275	0.199394	0	849	SAG-related sequence SRS36E
TGME49_212030	0.199655	0	3700	hypothetical protein
TGME49_264745	0.206883	0	375	hypothetical protein
TGME49_240365	0.211894	0	3700	hypothetical protein
TGME49_242390	0.219824	0	2144	enoyl-coa hydratase/isomerase family protein
TGME49_328300	0.226548	0	3700	hypothetical protein
TGME49_300700	0.233241	0	229	ribosomal protein S2, putative
TGME49_288960	0.25289	0	1183	hypothetical protein
TGME49_219670	0.254684	0	2220	zinc finger (CCCH type) motif-containing protein
TGME49_239650	0.255582	0	327	hypothetical protein
TGME49_297310	0.263004	0	3700	hypothetical protein
TGME49_218460	0.273517	0	2147	hypothetical protein

Gene ID	RNA-seq (TPM)	cumulative 5′-tag count (CPM)	window size (nt)	gene description
TGME49_293830	0.273931	0	1468	methyltransferase domain-containing protein
TGME49_324400	0.27588	0	200	hypothetical protein
TGME49_205402	0.33908	0	961	SNF2 family N-terminal domain-containing protein
TGME49_285770	0.354217	0	3700	hypothetical protein
TGME49_210235	0.354667	0	567	hypothetical protein
TGME49_274040	0.371614	0	789	hypothetical protein
TGME49_238480	0.373204	0	2468	SAG-related sequence SRS22D
TGME49_237900	0.392026	0	3700	OTU family cysteine protease
TGME49_323600	0.393483	0	1453	OTU family cysteine protease
TGME49_323700	0.393483	0	1453	OTU family cysteine protease
TGME49_323800	0.393483	0	1453	OTU family cysteine protease
TGME49_209485	0.398483	0	566	hypothetical protein
TGME49_218450	0.400793	0	612	hypothetical protein
TGME49_307620	0.404153	0	3441	hypothetical protein
TGME49_211370	0.414252	0	715	tetratricopeptide repeat-containing protein
TGME49_327800	0.416438	0	202	dynein-1-alpha heavy chain, flagellar inner arm I1 complex, putative
TGME49_254350	0.42493	0	3700	endo-1,3(4)-beta-glucanase
TGME49_233320	0.426387	0	435	hypothetical protein
TGME49_286782	0.432432	0	754	MAM domain-containing protein
TGME49_323200	0.474412	0	3700	OTU family cysteine protease
TGME49_318742	0.480134	0	272	hypothetical protein
TGME49_230602	0.49379	0	804	hypothetical protein
TGME49_302053	0.497098	0	3700	ribosomal protein S7
TGME49_302009	0.510664	0	210	ORF B
TGME49_322130	0.517485	0	3700	hypothetical protein
TGME49_269170	0.5302	0	596	hypothetical protein
TGME49_275798	0.554219	0	569	microneme protein, putative
TGME49_220060	0.598865	0	3700	hypothetical protein
TGME49_287510	0.628307	0	1006	aromatic amino acid hydrolase AAH1
TGME49_273468	0.660647	0	226	hypothetical protein
TGME49_240325	0.676097	0	3700	Toxoplasma gondii family E protein
TGME49_237894	0.67717	0	307	OTU family cysteine protease
TGME49_327000	0.69222	0	202	U5 small nuclear ribonucleoprotein component (U5 snRNP), putative
TGME49_201660	0.707784	0	3700	hypothetical protein
TGME49_283500	0.716251	0	2369	tetratricopeptide repeat-containing protein
TGME49_302057	0.764793	0	220	ribosomal protein S7
TGME49_316890	0.840968	0	3700	hypothetical protein
TGME49_271580	0.845979	0	358	Toxoplasma gondii family D protein
TGME49_321710	0.879631	0	1685	hypothetical protein
TGME49_289840	0.90463	0	480	hypothetical protein
TGME49_222975	0.940532	0	2071	hypothetical protein
TGME49_325100	0.94243	0	239	hypothetical protein
TGME49_324500	0.949843	0	3700	nuclear distribution protein C, putative
TGME49_315550	1.029425	0	3700	calcium binding egf domain-containing protein
TGME49_266330	1.058266	0	2984	Toxoplasma gondii family A protein
TGME49_214545	1.106045	0	1096	hypothetical protein
TGME49_210470	1.116715	0	1593	hypothetical protein
TGME49_272200	1.128544	0	3700	AGC kinase
TGME49_300608	1.136342	0	644	ribosomal protein L6, putative
TGME49_210095	1.143579	0	1644	hypothetical protein
TGME49_249280	1.164721	0	2623	hypothetical protein
TGME49_355200	1.16893	0	211	hypothetical protein
TGME49_244728	1.169015	0	1258	IQ calmodulin-binding motif domain-containing protein
TGME49_302003	1.217127	0	203	ORF E
TGME49_252090	1.244753	0	405	hypothetical protein
TGME49_206560	1.245626	0	3700	EF hand domain-containing protein
TGME49_316420	1.256356	0	297	leucine rich repeat-containing protein
TGME49_302005	1.287054	0	208	ORF F
TGME49_300660	1.29611	0	230	RNA polymerase B
TGME49_206550	1.382339	0	3700	hypothetical protein
TGME49_257460	1.480211	0	1350	hypothetical protein
TGME49_283702	1.492488	0	1047	FATC domain-containing protein
TGME49_301996	1.494764	0	340	ORF D
TGME49_327700	1.519009	0	497	hypothetical protein
TGME49_300606	1.572936	0	222	ribosomal protein S5, putative
TGME49_300631	1.653174	0	1408	ribosomal protein L4
TGME49_327900	1.673563	0	200	hypothetical protein
TGME49_324000	1.822461	0	3700	dynein heavy chain, n-terminal region 2 protein
TGME49_328400	1.868077	0	201	aminotransferase, class V family protein
TGME49_231620	1.877585	0	1152	hypothetical protein
TGME49_300690	1.900463	0	201	RNA polymerase C2, putative
TGME49_269350	1.974316	0	926	hypothetical protein
TGME49_255860	2.037263	0	243	hypothetical protein
TGME49_328600	2.20116	0	3700	Isoleucine-tRNA ligase
TGME49_233065	2.2405	0	1594	3'5'-cyclic nucleotide phosphodiesterase domain-containing protein
TGME49_254320	2.269332	0	1497	hypothetical protein
TGME49_300604	2.310192	0	216	ribosomal protein L36
TGME49_322120	2.340336	0	536	hypothetical protein
TGME49_312435	2.370091	0	3700	hypothetical protein
TGME49_207780	2.380868	0	3700	hypothetical protein
TGME49_328000	2.449515	0	612	hypothetical protein
TGME49_300615	2.612814	0	260	ribosomal protein S3, putative
TGME49_300618	2.652662	0	219	ribosomal protein S19
TGME49_269930	2.683562	0	3700	calcium binding egf domain-containing protein
TGME49_327300	2.797956	0	805	Toxoplasma gondii family A protein
TGME49_287490	2.854668	0	3700	hypothetical protein
TGME49_307790	2.880539	0	414	hypothetical protein
TGME49_219840	2.902313	0	3700	hypothetical protein
TGME49_322200	2.913231	0	202	apocytochrome b, putative
TGME49_300680	2.939306	0	219	RNA polymerase C1, putative
TGME49_210478	3.025129	0	1471	hypothetical protein
TGME49_251857	3.082531	0	406	hypothetical protein
TGME49_325000	3.240893	0	321	hypothetical protein
TGME49_205430	3.260328	0	2300	isovaleryl-CoA dehydrogenase
TGME49_300612	3.402444	0	204	ribosomal protein S17
TGME49_244710	3.607221	0	474	hypothetical protein

Gene ID	RNA-seq (TPM)	cumulative 5′-tag count (CPM)	window size (nt)	gene description
TGME49_210482	3.691844	0	431	hypothetical protein
TGME49_328800	3.716701	0	3700	hypothetical protein
TGME49_313322	3.959923	0	223	hypothetical protein
TGME49_302060	4.084246	0	258	elongation factor tu, apicoplast, putative
TGME49_243482	4.096481	0	1251	dynein heavy chain, putative
TGME49_237810	4.235874	0	589	hypothetical protein
TGME49_324900	4.339128	0	3700	hypothetical protein
TGME49_302007	4.449818	0	512	ribosomal protein L11, putative
TGME49_328500	4.491915	0	3700	hypothetical protein
TGME49_263450	4.852167	0	373	hypothetical protein
TGME49_325500	4.873367	0	201	ATP-binding Mrp/Nbp35 family protein
TGME49_326900	4.906435	0	3700	hypothetical protein
TGME49_325600	4.991439	0	3700	hypothetical protein
TGME49_325700	4.998834	0	3700	ATP-binding protein
TGME49_326400	5.243804	0	202	hypothetical protein
TGME49_326700	5.522953	0	368	DNA-directed RNA polymerase, putative
TGME49_300621	5.530445	0	221	ribosomal protein L2
TGME49_327500	5.699553	0	3700	hypothetical protein
TGME49_328100	6.24049	0	200	hypothetical protein
TGME49_276820	6.522248	0	289	hypothetical protein
TGME49_327600	6.720608	0	200	hypothetical protein
TGME49_324700	6.794656	0	200	hypothetical protein
TGME49_292600	7.01509	0	1953	hypothetical protein
TGME49_300601	7.618827	0	203	ribosomal protein S11
TGME49_302000	7.879637	0	242	chaperone clp, putative
TGME49_242250	8.136186	0	2921	rhoptry kinase family protein ROP19B
TGME49_327400	8.748797	0	202	dynein heavy chain family protein
TGME49_227995	9.454196	0	633	hypothetical protein
TGME49_251958	9.807015	0	2993	SAG-related sequence SRS59B
TGME49_218890	9.807849	0	3700	hypothetical protein
TGME49_209780	10.014587	0	3700	hypothetical protein
TGME49_323900	10.958092	0	400	hypothetical protein
TGME49_293030	11.753446	0	341	hypothetical protein
TGME49_326600	12.238255	0	202	hypothetical protein
TGME49_325400	13.729887	0	861	ribosomal L37ae family protein
TGME49_301216	14.779078	0	222	endonuclease/exonuclease/phosphatase family protein
TGME49_314770	14.877214	0	1422	tRNA (guanine-N1)-methyltransferase
TGME49_293650	15.454359	0	3700	hypothetical protein
TGME49_327100	15.823336	0	200	U5 small nuclear ribonuclear protein, putative
TGME49_200295	15.910655	0	249	hypothetical protein
TGME49_326000	16.936119	0	3700	RNA binding protein, putative
TGME49_326500	17.856398	0	3700	hypothetical protein
TGME49_320530	18.552643	0	3338	hypothetical protein
TGME49_324300	20.34614	0	228	transitional endoplasmic reticulum ATPase, putative
TGME49_256220	22.279211	0	3700	hypothetical protein
TGME49_292290	23.866205	0	1325	hypothetical protein
TGME49_325900	24.027069	0	3700	zinc finger (CCCH type) motif-containing protein
TGME49_210975	24.095633	0	3700	hypothetical protein
TGME49_249660	24.702267	0	2547	hypothetical protein
TGME49_254340	28.324579	0	305	hypothetical protein
TGME49_248240	29.842287	0	2316	leucine rich repeat-containing protein
TGME49_254250	30.762758	0	222	PRELI family protein
TGME49_322210	34.705959	0	1215	apocytochrome b, putative
TGME49_220650	37.683559	0	538	hypothetical protein
TGME49_250670	46.296749	0	3700	hypothetical protein
TGME49_285300	46.979683	0	1417	hypothetical protein
TGME49_327200	47.402206	0	202	rhoptry neck protein, putative
TGME49_326100	48.88052	0	201	dynamin gtpase
TGME49_325300	52.279186	0	3700	proteasome subunit alpha type, putative
TGME49_229690	52.36055	0	248	autophagy-related protein 7 atg7, putative
TGME49_242230	55.331512	0	2701	rhoptry kinase family protein ROP29
TGME49_249535	56.958549	0	301	hypothetical protein
TGME49_260205	59.530392	0	242	hypothetical protein
TGME49_209610	59.782486	0	1834	oocyst wall protein OWP2
TGME49_329800	64.799286	0	240	hypothetical protein
TGME49_330000	71.770958	0	3700	cytochrome b
TGME49_322600	75.812347	0	3700	hypothetical protein
TGME49_255990	80.784508	0	3700	hypothetical protein
TGME49_324100	92.818054	0	3700	hypothetical protein
TGME49_323300	99.49781	0	200	myosin-light-chain kinase
TGME49_326800	102.856682	0	3700	S-Adenosyl homocysteine hydrolase
TGME49_287440	103.432396	0	1914	hypothetical protein
TGME49_220640	113.349426	0	505	hypothetical protein
TGME49_323400	113.458412	0	661	cytochrome c oxidase subunit iii subfamily protein
TGME49_255060	117.900536	0	3700	cytochrome b(N-terminal)/b6/petB subfamily protein
TGME49_324800	140.110291	0	3700	tryptophanyl-tRNA synthetase
TGME49_287430	141.632324	0	477	hypothetical protein
TGME49_322000	149.185608	0	3700	myosin-light-chain kinase
TGME49_318310	152.758621	0	295	transketolase
TGME49_328700	536.867615	0	3700	hypothetical protein
TGME49_219110	0	0	1295	hypothetical protein
TGME49_230605	0	0	803	hypothetical protein
TGME49_230670	0	0	330	Tubulin-tyrosine ligase family protein
TGME49_233270	0	0	1679	MORN repeat-containing protein
TGME49_240920	0	0	3700	hypothetical protein
TGME49_243130	0	0	2169	Toxoplasma gondii family A protein
TGME49_243150	0	0	1204	Toxoplasma gondii family A protein
TGME49_259290	0	0	3700	SAG-related sequence SRS26C
TGME49_263250	0	0	1005	Pb-fam-5 protein
TGME49_269110	0	0	3700	ornithine aminotransferase, mitochondrial precursor, putative
TGME49_271220	0	0	1037	hypothetical protein
TGME49_278310	0	0	432	hypothetical protein
TGME49_278690	0	0	997	cpw-wpc domain-containing protein
TGME49_280070	0	0	3700	hypothetical protein
TGME49_283470	0	0	3700	Kazal-type serine protease inhibitor domain-containing protein
TGME49_283490	0	0	3700	Alpha-amylase AMY3, putative

Gene ID	RNA-seq (TPM)	cumulative 5′-tag count (CPM)	window size (nt)	gene description
TGME49_286100	0	0	532	hypothetical protein
TGME49_298090	0	0	382	Toxoplasma gondii family B protein
TGME49_302150	0	0	456	ATPase, AAA family protein
TGME49_302450	0	0	234	TFIIH basal transcription factor complex helicase XPB subunit, putative
TGME49_303050	0	0	1926	zinc finger, c2h2 type family protein
TGME49_303450	0	0	270	coatomer gamma subunit
TGME49_319988	0	0	283	hypothetical protein
TGME49_322900	0	0	690	hypothetical protein
TGME49_323500	0	0	402	hypothetical protein
TGME49_329500	0	0	3700	hypothetical protein
TGME49_329700	0	0	204	SAG-related sequence SRS26J

Table S3. Manual inspection of loci randomly-sampled from candidates for alternative TSS usage

Gene ID	TSS shift* (nt)	assessment	uAUG gain*	uORF gain*
TGME49_280800	52	putative alternative TSS usage (stage-dependent)	0	0
TGME49_216240	-2030	putative alternative TSS usage (stage-dependent)	21	13
TGME49_232020	206	putative alternative TSS usage (stage-dependent)	-6	-3
TGME49_239930	-400	putative alternative TSS usage (stage-dependent)	4	4
TGME49_262560	181	putative alternative TSS usage (stage-dependent)	0	0
TGME49_282220	-593	putative alternative TSS usage (stage-dependent)	11	4
TGME49_215010	130	putative alternative TSS usage (stage-dependent)	0	0
TGME49_311650	129	putative alternative TSS usage (stage-dependent)	0	0
TGME49_270320	772	putative alternative TSS usage (stage-dependent)	-5	-3
TGME49_265060	1412	putative alternative TSS usage (stage-dependent)	-24	-12
TGME49_219210	292	putative alternative TSS usage (stage-dependent)	-3	-1
TGME49_295450	-637	putative alternative TSS usage (stage-dependent)	9	6
TGME49_286510	272	putative alternative TSS usage (stage-dependent)	-2	-1
TGME49_263570	410	putative alternative TSS usage (stage-dependent)	-3	-2
TGME49_283878	-440	putative alternative TSS usage (stage-dependent)	7	4
TGME49_232000	676	putative alternative TSS usage (stage-dependent)	NA	NA
TGME49_219320	-197	putative alternative TSS usage (stage-independent)	1	1
TGME49_315420	47	putative alternative TSS usage (stage-independent)	0	0
TGME49_239320	-209	putative alternative TSS usage (stage-independent)	NA	NA
TGME49_230490	64	putative alternative TSS usage (stage-independent)	0	0
TGME49_225580	59	putative alternative TSS usage (stage-independent)	-2	-1
TGME49_231590	-259	putative alternative TSS usage (stage-independent)	4	4
TGME49_299200	247	putative alternative TSS usage (stage-independent)	0	0
TGME49_306440	-771	putative alternative TSS usage (stage-independent)	15	11
TGME49_215990	-2923	putative alternative TSS usage (stage-independent)	30	19
TGME49_285190	65	putative alternative TSS usage (stage-independent)	-1	-1
TGME49_282210	-2890	capture of pervasive transcription from neighboring genes	NA	NA
TGME49_205680	-1645	capture of pervasive transcription from neighboring genes	NA	NA
TGME49_321630	-2792	capture of pervasive transcription from neighboring genes	NA	NA
TGME49_247740	75	capture of pervasive transcription from neighboring genes	NA	NA
TGME49_257330	-2289	capture of pervasive transcription from neighboring genes	NA	NA
TGME49_249360	-2204	capture of pervasive transcription from neighboring genes	NA	NA
TGME49_259860	-1104	capture of pervasive transcription from neighboring genes	NA	NA
TGME49_258740	-2867	capture of pervasive transcription from neighboring genes	NA	NA
TGME49_262410	-2294	capture of pervasive transcription from neighboring genes	NA	NA
TGME49_219682	-1210	capture of pervasive transcription from neighboring genes	NA	NA
TGME49_207200	-2797	capture of pervasive transcription from neighboring genes	NA	NA
TGME49_297980	-263	capture of pervasive transcription from neighboring genes	NA	NA
TGME49_293340	-1490	capture of pervasive transcription from neighboring genes	NA	NA
TGME49_215470	-3307	capture of pervasive transcription from neighboring genes	NA	NA
TGME49_297080	2652	capture of pervasive transcription from neighboring genes	NA	NA
TGME49_218390	-3378	gene model incorrect	NA	NA
TGME49_270260	151	gene model incorrect	NA	NA
TGME49_313220	2179	gene model incorrect	NA	NA
TGME49_233960	-1266	gene model incorrect	NA	NA
TGME49_272680	1895	gene model incorrect	NA	NA
TGME49_230970	996	gene model incorrect	NA	NA
TGME49_309190	-855	complex locus	NA	NA
TGME49_222340	264	complex locus	NA	NA
TGME49_252065	-1255	low counts / noise	NA	NA

*relative to Tz prediction
